# Species-targeted sorting and cultivation of commensal bacteria from the gut microbiome using flow cytometry under anaerobic conditions

**DOI:** 10.1186/s40168-021-01206-7

**Published:** 2022-02-03

**Authors:** Samuel Bellais, Mélanie Nehlich, Maryne Ania, Aurore Duquenoy, Wilfrid Mazier, Ger van den Engh, Jan Baijer, Nicole Simone Treichel, Thomas Clavel, Ilia Belotserkovsky, Vincent Thomas

**Affiliations:** 1 BIOASTER, 28 rue du Docteur Roux, 75015 Paris, France; 2 MaaT Pharma, Lyon, France; 3 YSOPIA Bioscience, Bordeaux, France; 4 Marine Cytometry, Concrete, WA 98237 USA; 5 Commissariat à l’Energie Atomique et aux Energies Alternatives, Département de la Recherche Fondamentale, Institut de Biologie François Jacob, Institut de Radiobiologie Cellulaire et Moléculaire, Fontenay-aux-Roses, France; 6 Functional Microbiome Research Group, Institute of Medical Microbiology, University Hospital of RWTH, Aachen, Germany

**Keywords:** Microbiota, *Faecalibacterium*, *Christensenella*, Anaerobic, Flow cytometry, Sorting, Culturomics

## Abstract

**Background:**

There is a growing interest in using gut commensal bacteria as “next generation” probiotics. However, this approach is still hampered by the fact that there are few or no strains available for specific species that are difficult to cultivate. Our objective was to adapt flow cytometry and cell sorting to be able to detect, separate, isolate, and cultivate new strains of commensal species from fecal material. We focused on the extremely oxygen sensitive (EOS) species *Faecalibacterium prausnitzii* and the under-represented, health-associated keystone species *Christensenella minuta* as proof-of-concept.

**Results:**

A BD Influx® cell sorter was equipped with a glovebox that covered the sorting area. This box was flushed with nitrogen to deplete oxygen in the enclosure. Anaerobic conditions were maintained during the whole process, resulting in only minor viability loss during sorting and culture of unstained *F. prausnitzii* strains ATCC 27766, ATCC 27768, and DSM 17677. We then generated polyclonal antibodies against target species by immunizing rabbits with heat-inactivated bacteria. Two polyclonal antibodies were directed against *F. prausnitzii* type strains that belong to different phylogroups, whereas one was directed against *C. minuta* strain DSM 22607. The specificity of the antibodies was demonstrated by sorting and sequencing the stained bacterial fractions from fecal material. In addition, staining solutions including LIVE/DEAD™ BacLight™ Bacterial Viability staining and polyclonal antibodies did not severely impact bacterial viability while allowing discrimination between groups of strains. Finally, we combined these staining strategies as well as additional criteria based on bacterial shape for *C. minuta* and were able to detect, isolate, and cultivate new *F. prausnitzii* and *C. minuta* strains from healthy volunteer’s fecal samples.

**Conclusions:**

Targeted cell-sorting under anaerobic conditions is a promising tool for the study of fecal microbiota. It gives the opportunity to quickly analyze microbial populations, and can be used to sort EOS and/or under-represented strains of interest using specific antibodies, thus opening new avenues for culture experiments.

Video abstract

**Supplementary Information:**

The online version contains supplementary material available at 10.1186/s40168-021-01206-7.

## Background

With the availability of next-generation sequencing technologies that allow high-throughput analysis of the composition and function of complex microbial ecosystems, the field of microbiome research has grown rapidly in recent years, and countless associations have been reported between microbiota composition and specific health conditions. This is especially true for the human gut ecosystem, for which microbial signatures have been associated with metabolic syndrome, inflammatory bowel diseases (IBD), and response to cancer immunotherapy to mention just a few. This offers new fundamental and applied research avenues, with the ultimate goal to develop new, complementary tools for treating these conditions [1–3]. In particular, 16S rRNA gene amplicon or shotgun metagenomic analysis conducted on fecal samples collected from cohorts of patients vs. controls highlighted decreased occurrence of several commensal bacterial species in pathological conditions [4]. There is thus a growing interest in using cultured, well-characterized strains to complement deficiencies in the gut microbiota, referred to as “next-generation probiotics” (NGP) [5].

This is the case for *Faecalibacterium prausnitzii*, which accounts for about 5‑10% of dominant microbial communities within the healthy gut microbiota [6], and has been associated with a number of favorable outcomes in various pathologies including lower risk of postoperative recurrence of ileal Crohn’s disease [7] and an improved response to immune check point blockers [8, 9]. The phylogeny of *F. prausnitzii* is complex, comprising at least 3 different phylogroups, and possibly represents several species that remain to be described taxonomically [10–12]. Relative proportions of the different phylogroups in one same individual seem to vary depending on specific disease condition, with phylogroup IIb strains being depleted in Crohn’s disease patients [13, 14]. It has consequently been proposed to use corresponding relative abundances as disease biomarker [15].

Other NGP candidates can be found within the family *Christensenellaceae* [16]. Relative abundancy of these heritable bacteria is inversely correlated to host body mass index and the type species *Christensenella minuta* has been demonstrated to reduce weight gain in germ-free mice colonized with fecal microbiota collected from obese individuals [17]. Recently, it has also been reported that *C. minuta* DSM33407 protected from diet-induced obesity and regulated associated metabolic markers such as glycemia and leptin in a diet-induced obesity mouse model [18].

In this context, and knowing that specific biological properties of gut bacteria, including host beneficial properties, can vary significantly from one strain to another [19], there is a major interest in building collections of different commensal strains of target species of interest identified via NGS studies. However, this approach is still hampered by the fact that retrieving target species from clinical samples (usually fecal material) can be difficult. Extreme oxygen sensitivity (EOS) (*F. prausnitzii*) or under-representation of the target species in the community (*C. minuta*) can be important limitations, with the addition of specific nutritional requirements rendering target species difficult to cultivate in synthetic media. Flow cytometry (FCM) coupled with cell-sorting has the potential to circumvent most if not all these limitations. With constantly increasing technological performances, FCM can be used for bacterial or even viral cell populations’ analysis with or without subsequent sorting [20, 21]. With the objective to use FCM and cell-sorting to analyze, sort, and cultivate bacterial species of interest from fecal samples, we adapted a cell sorter and associated workflow to conduct sorting experiments under strictly anaerobic conditions. We then evaluated the impact of sorting as well as non-specific and specific staining methods on the viability of several representative strains of the EOS species *F. prausnitzii*. To test the feasibility of the specific targeting of bacteria using anaerobic sorting, we isolated new *F. prausnitzii* strains from fecal samples using antibodies raised against available reference strains. Finally, we applied this method to sort and cultivate novel strains of *C. minuta*, which is more tolerant to oxygen exposure but usually found at low abundance (in contrast to *F. prausnitzii*), demonstrating the potential of this approach.

## Results

### Evaluation of polyclonal antibodies against reference strains of *F. prausnitzii* and *Christensenella* spp.

**Fig. 1:**
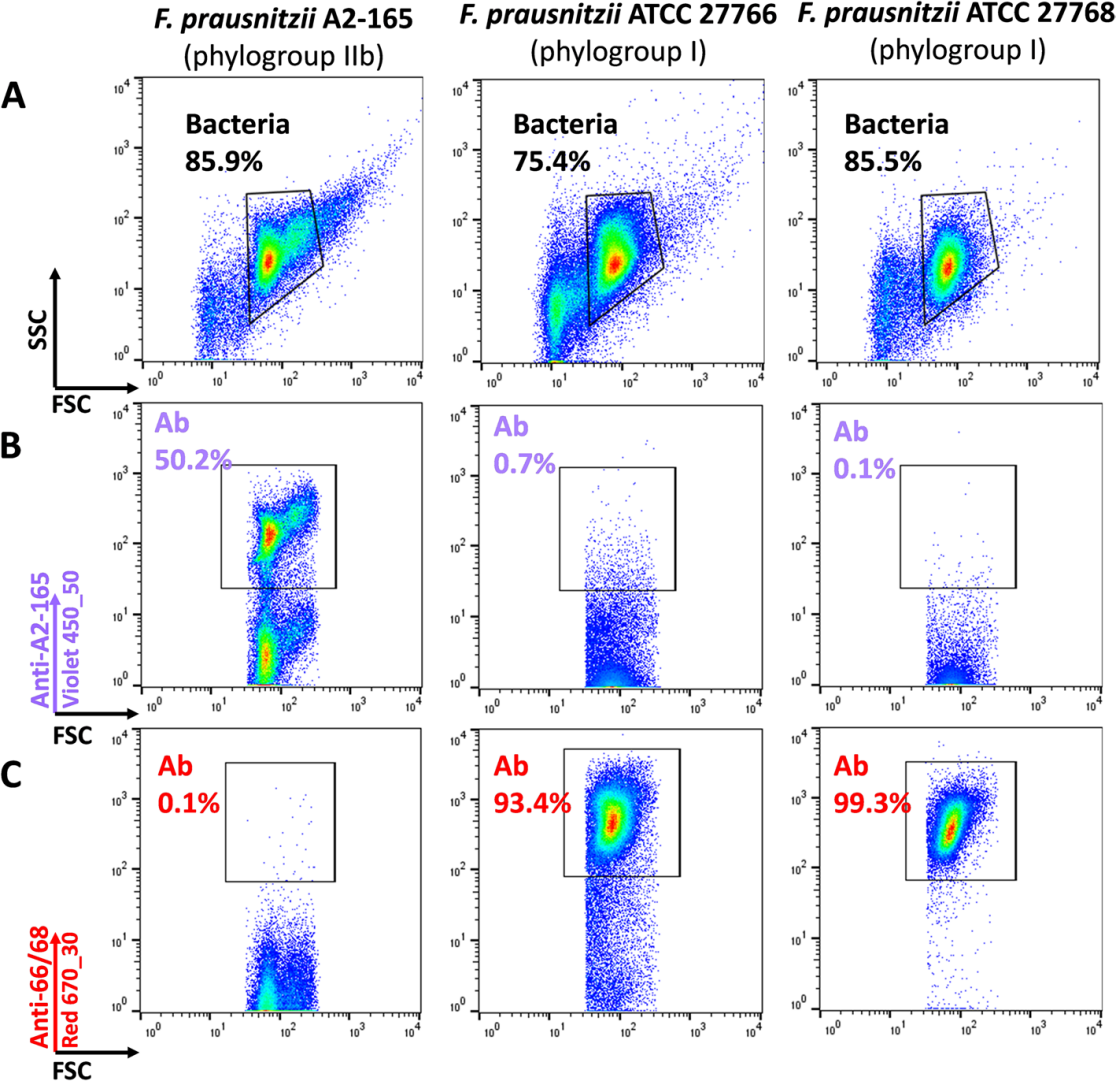
Representative staining of *F. prausnitzii* reference strains. Fresh cultures of the three strains: DSM-17677 (A2-165), ATCC-27766, and ATCC-27768 were analyzed after staining with polyclonal antibodies. The measurements were based on gated events corresponding to the vast majority of bacteria in the suspensions (**A**). Proportions of bacteria stained with polyclonal antibodies generated with strain A2-165 (**B**), or with polyclonal antibodies generated with both ATCC strains (**C**) were then analyzed. These experiments were repeated 3 times

A recent publication suggested that the species *F. prausnitzii* comprises at least two phylogroups whose contribution to human health might be different [10]. Therefore, we used representatives of both phylogroups to generate polyclonal antibodies: strain DSM 17677 (A2-165, phylogroup IIb) and a mix of the closely related strains ATCC 27766 and ATCC 27768 (phylogroup I). Antibodies raised against the ATCC strains were consistently effective in detecting > 90% of target bacteria. They showed cross-reactivity with strain A2-165 but the intensity of the staining was much lower, thus allowing the definition of a phylogroup-specific gating region (Fig. 1 and Additional files 1 and 3). Staining efficiency was more variable for antibodies directed against strain A2-165, with the proportion of stained bacteria ranging from 50% to > 90% depending on the experiment. These antibodies also slightly reacted with the ATCC strains but here again, a phylogroup-specific gating region could easily be defined (Fig. 1 and Additional files 1 and 3). We did not observe any fluorescence of unstained *F. prausnitzii* cells when exciting with the Violet 405 (450/50 nm) or Red 670 (670/30 nm) lasers whereas significant auto-fluorescence was observed for the 3 different strains when exciting with the Blue 540/30 nm laser (Additional file 1). However, in this case, the signal was still 1 to 2 log lower than the signal used for gating live cells after staining with SYTO 9 contained in the LIVE/DEAD™ kit (Additional file 3), so auto-fluorescence in that channel is not likely to interfere with the viability staining.

**Fig. 2:**
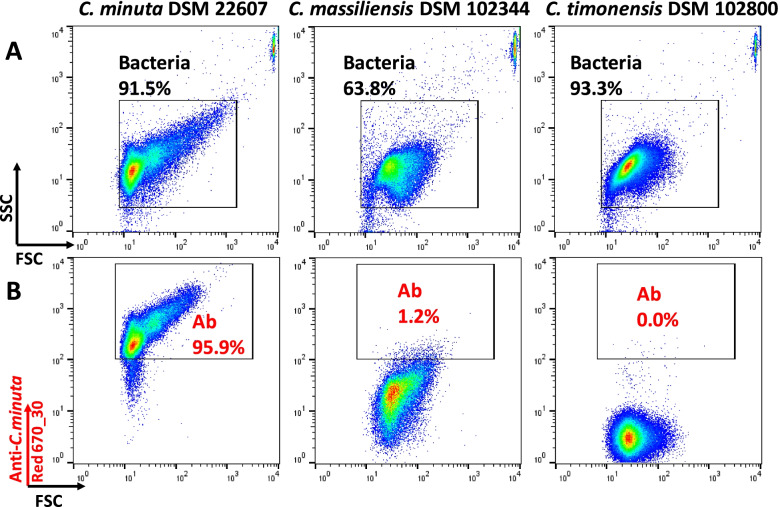
Representative staining of *Christensenellaceae* reference strains. Fresh cultures of *Christensenella minuta* DSM 22607, “Christensenella massiliensis” DSM 102344 and “Christensenella timonensis” DSM 102800 were analyzed. The measurements were made only with gated events corresponding to the vast majority of bacteria in the suspensions (**A**). Proportions of bacteria stained with polyclonal antibodies generated with *C. minuta* DSM 22607 strain were then analyzed (**B**). These experiments were repeated 3 times

We also generated polyclonal antibodies by injecting rabbits with heat-inactivated bacteria of the publicly available *C. minuta* strain DSM 22607. These antibodies were then tested against a pure culture of the parental strain and the related species “Christensenella massiliensis” and “Christensenella timonensis.” Nearly all cells within the pure culture of *C. minuta* were indeed stained (Fig. 2 and Additional files 2 and 3). Limited cross-reactivity was observed with “C. massiliensis,” for which, depending on the experiment, 1.2 to 14.3% of the bacteria were stained by *C. minuta* antibodies (Fig. 2 and Additional file 2). There was no cross-reactivity with “C. timonensis.”

### Impact of staining and anaerobic sorting on *F. prausnitzii* and *C. minuta viability*

**Fig. 3:**
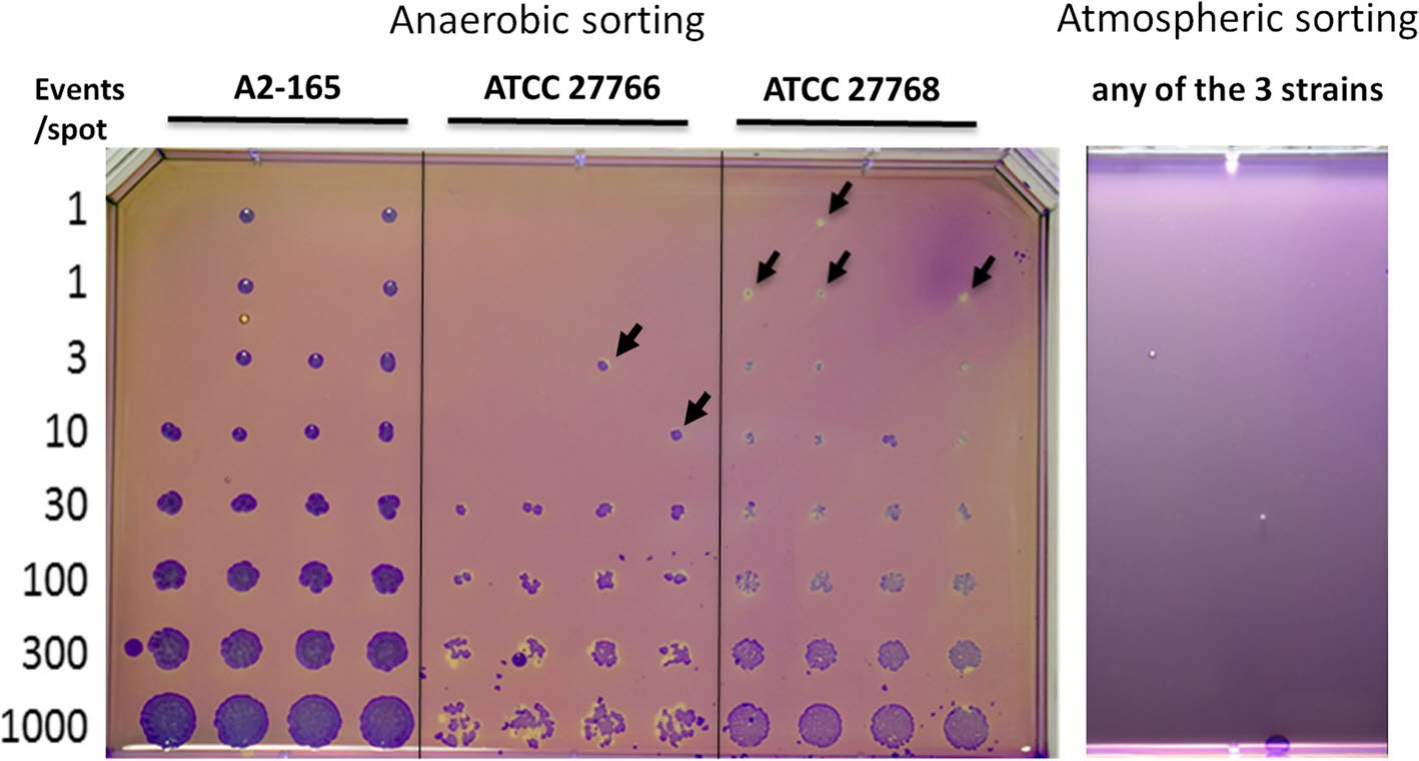
Impact of sorting under anaerobic or atmospheric conditions on *F. prausnitzii* recovery. Fresh cultures of the 3 *F.prausnitzii* reference strains were sorted on mGAM plates in anaerobic (left) or atmospheric (right) conditions. Increasing (1 to 1000) amounts of bacteria were sorted and plated on each spot. Colonies were stained with crystal violet for better visualization of tiny colonies (black arrows). Experiments were repeated at least 3 times

We then tested the effect of the sorting procedures on viability of the EOS species *F. prausnitzii* [22] by comparing two conditions: (i) sorting performed under anaerobic conditions, (ii) sorting performed under normal atmosphere [23]. The recovery of unlabeled *F. prausnitzii* after sorting under anaerobic conditions was approximately 20% for the three tested strains, while no colony could be observed if the sorting was performed under normal atmosphere (Fig. 3).

**Fig. 4:**
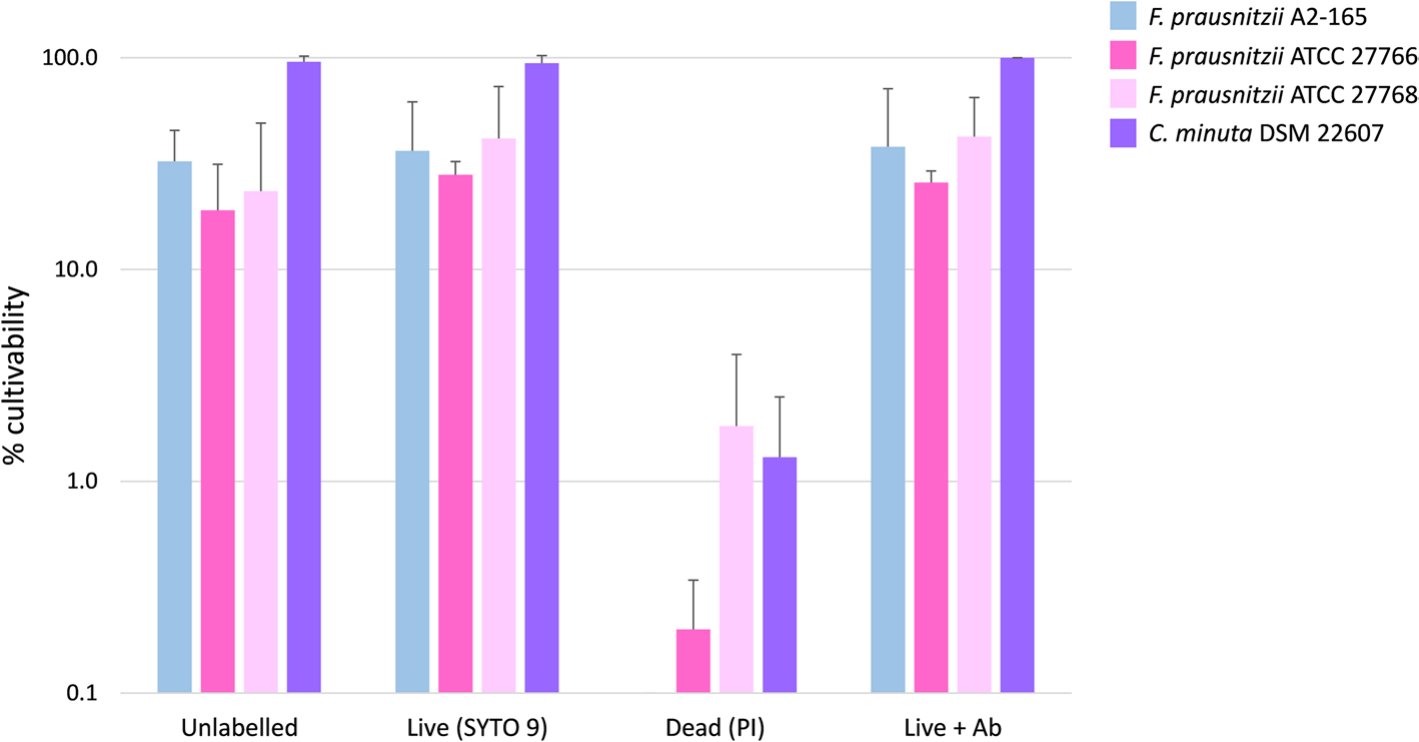
Fractions of bacteria recovered in culture after various staining. Fresh cultures of the three *F. prausnitzii* and *C. minuta* reference strains were stained with SYTO 9 and propidium iodide, and with specific antibodies and were then sorted in increasing amounts on mGAM plates to calculate the percentage of cultivability. Experiments were performed two times in triplicates

We then evaluated the effect of different staining methods (SYTO 9 and propidium iodide in the LIVE/DEAD™ *Bac*Light™ Bacterial Viability Kit and the 4 strain specific antibodies) on cultivability compared to unstained bacteria (see sorting gates in Additional file 3). These experiments showed no significant impact of any of the tested staining on *F. prausnitzii* cultivability during sorting in anaerobic conditions (Fig. 4). Several colonies were still observed after anaerobic sorting and cultivation of the propidium iodide (PI)-stained fraction, corresponding to approximately 0.1 to 1% of cultivable bacteria in this fraction (Fig. 4). The cultivability of *C. minuta* was very high, and remained unaffected after LIVE/DEAD™ and antibody staining.

#### Unstained and pre-immune controls

We did not detect any auto-fluorescence in the Red 670/30 nm channel for samples collected from healthy volunteers (HV) 1 to 4. Additional signal observed when the same samples were stained with the pre-immune fluorescent antibodies was limited, with percentages of stained events ranging from 0.01 to 0.21% (Additional file 4). The signal detected after staining with Alexa Fluor™ 647-antibodies directed against the ATCC strains of *F. prausnitzii* was clearly distinguishable from the background (Additional file 4, HV1 and HV4). The situation was markedly different in the 450/50 nm channel, with auto-fluorescent events being clearly detected as distinct populations accounting for 0.03 to 1.96% of the events in the “bacteria” gate (Additional file 4). However, samples in which events were detected after staining with Alexa Fluor™ 405-antibodies directed against *F. prausnitzii* A2-165 displayed populations that could clearly be distinguished from the background auto-fluorescence from unstained bacteria (Additional file 4, HV1 and HV4).

#### Evaluation of antibodies’ specificity and enrichment rates for fractions sorted after staining with polyclonal antibodies and for the 450 nm-auto-fluorescent fraction

**Fig. 5:**
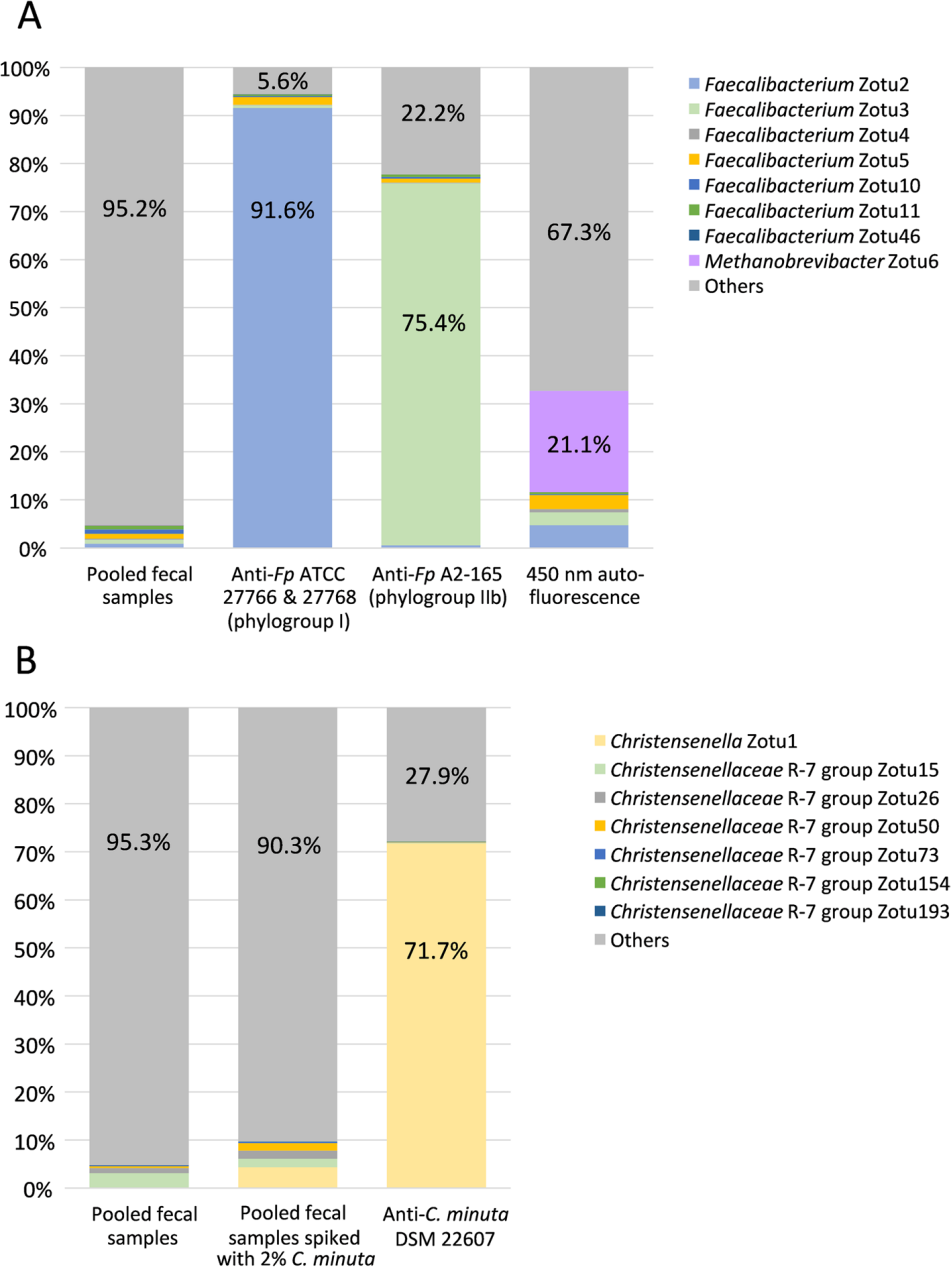
Selective enrichment and 16S rRNA gene amplicon analysis of bacterial populations sorted after staining of pooled fecal samples HV2 + HV4 with polyclonal antibodies directed against *F. prausnitzii* ATCC 27766 + 27768 and *F. prausnitzii* A2-165 (**A**), and with polyclonal antibodies directed against *C. minuta* DSM 22607 (**B**). Bacteria presenting auto-fluorescence in the 450/50 nm channel after excitation with the 405 nm laser were also sorted and analyzed by sequencing (**A**). One million bacteria were sorted for each population to be sequenced, and a 100% identity threshold corresponding to the zero radius OTU (zOTU) definition was used to delineate OTUs [24]. These experiments were performed once for *F. prausnitzii* (**A**) and once for *C. minuta* (**B**)

**Fig. 6:**
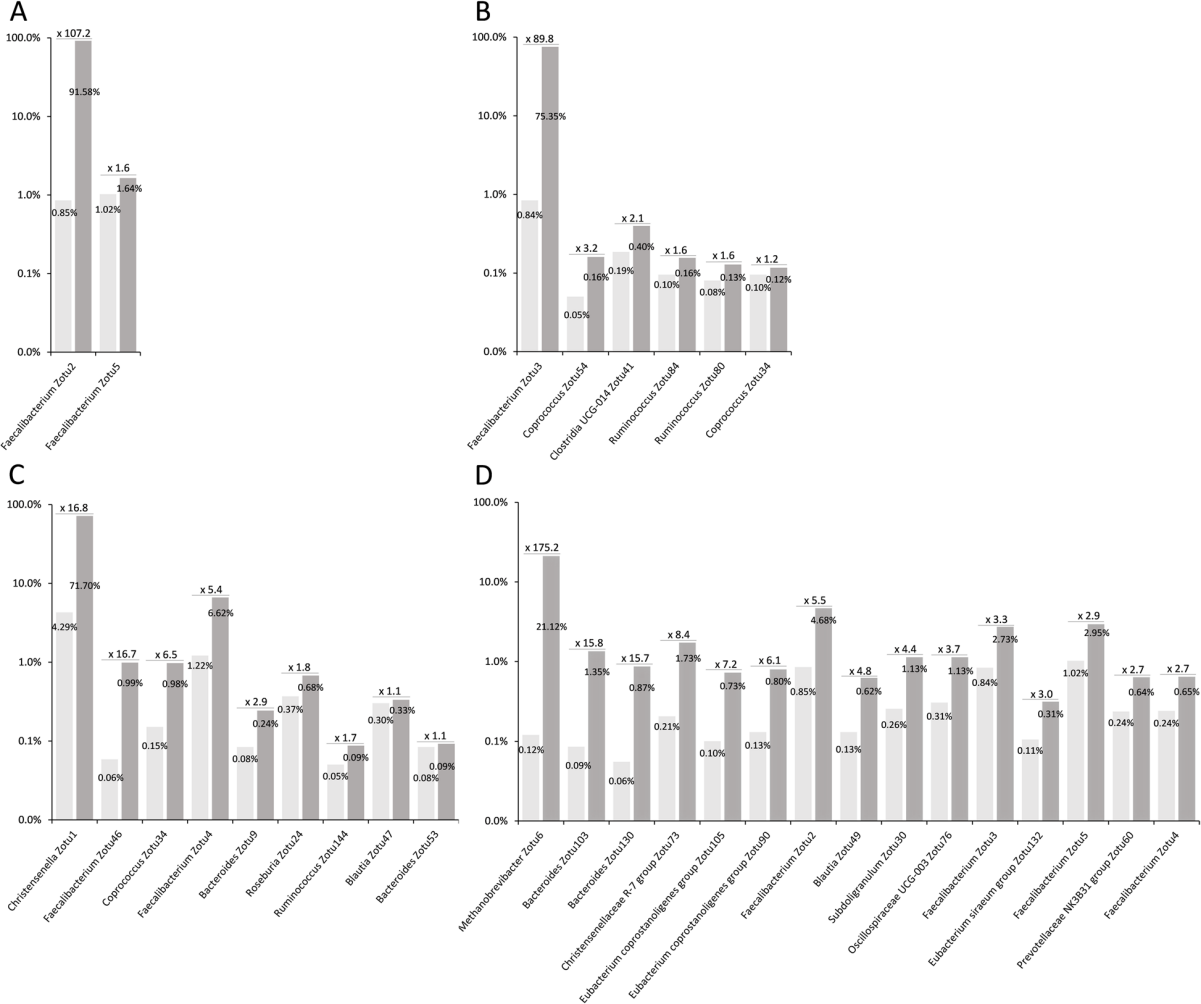
Enrichment rates of specific phylogenetic groups in the populations sorted after staining with the polyclonal antibodies directed against *F. prausnitzii* ATCC 27766 + ATCC 277668 (**A**), *F. prausnitzii* A2-165 (**B**), *C. minuta* DSM 22607 (**C**), and in bacterial population presenting auto-fluorescence in the 450/50 nm channel after excitation with the 405 nm laser (**D**). Rates were calculated by dividing normalized numbers of sequences affiliated to each zOTU in the sorted fractions (dark gray) by normalized numbers of sequences affiliated to the same zOTU in the raw material (light gray). Only groups with a least 0.05% affiliated sequences in the 16S rRNA gene amplicon repertoire of pooled fecal samples and for which enrichment rate is ≥ 1 were considered, and a maximum of 15 most enriched groups are shown for each antibody. These experiments were performed once for *F. prausnitzii* and autofluorescent bacteria (**A**, **B**, and **D**), and once for *C. minuta* (**C**)

As shown in Fig. 5A, we observed a very substantial enrichment in the sorted fractions, with 91.6% of the 16S rRNA gene amplicon sequences being annotated as *F. prausnitzii* in the fraction sorted using the antibody directed against strains ATCC 27766 and ATCC 27768, and 75.4% in the fraction sorted using the antibody directed against strain A2-165. The enrichment rates calculated on the basis of the normalized sequences were of the order of 100 for both antibodies and there was no significant enrichment of other zOTUs in the *F. prausnitzii*-sorted fractions (Fig. 6A and B).

Since auto-fluorescent events were detected in samples collected from HV4 when exciting with the 405 nm laser, we also sorted and then performed 16S rRNA gene amplicon sequencing and analysis of the sorted fraction from HV2+HV4 mix. When excluding zOTUs to which less than 0.05% of the normalized sequences were affiliated in the original fecal material, the most enriched bacteria in the sorted fraction were affiliated to the *Methanobacteriaceae* (Figs. 5A and 6D). Several zOTUs affiliated to genera *Bacteroides*, *Eubacterium*, and *Faecalibacterium*, and to the *Christensenellaceae* R-7 group were also enriched by more than 5 times in the sorted fraction compared to the original pooled material (Fig. 6D).

For the fraction sorted from the pool of 2 samples spiked at approx. 2% with the *C. minuta* collection strain (see Additional file 5 for sorting gates), 71.7% of the sequences were indeed annotated as *C. minuta* (Fig. 5B). The enrichment rate calculated on the basis of the normalized sequences was of 16.8, and it was of 16.7 for a zOTU affiliated to the genus *Faecalibacterium* (Fig. 6C), suggesting that these antibodies can potentially cross-react with species other than *C. minuta*.

### Use of polyclonal antibodies for sorting and cultivation of new *F. prausnitzii strains* from fecal material

**Fig. 7:**
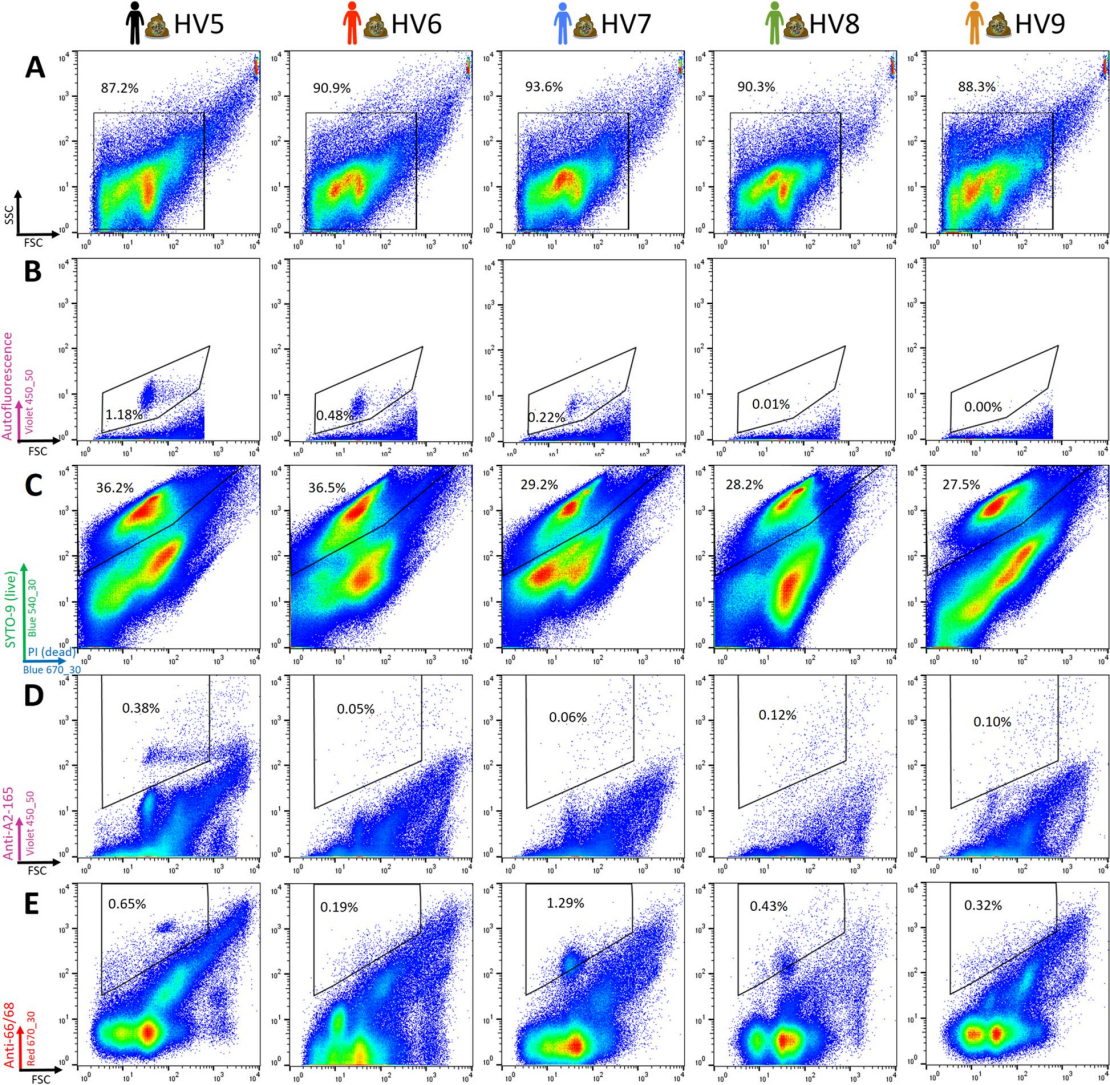
Flow cytometry analysis of fecal microbiota collected from 5 healthy volunteers (HV5 to HV9). A first gate was defined to select events that likely correspond to bacteria (**A**). The second gate (**B**) was defined to exclude events that were auto-fluorescent in the 450/50 nm channel after excitation with the 405 nm laser. The third gate (**C**) was defined to select bacteria considered as live after staining with the LIVE/DEAD™ *Bac*Light™ Bacterial Viability Kit. Among them, 240 events stained with anti-*F. prausnitzii* A2-165 (**D**) or anti-*F. prausnitzii* ATCC 27766 + ATCC 27768 (**E**) antibodies were selected for sorting and cultivation. These experiments were performed once for each of the 5 healthy volunteers

**Fig. 8:**
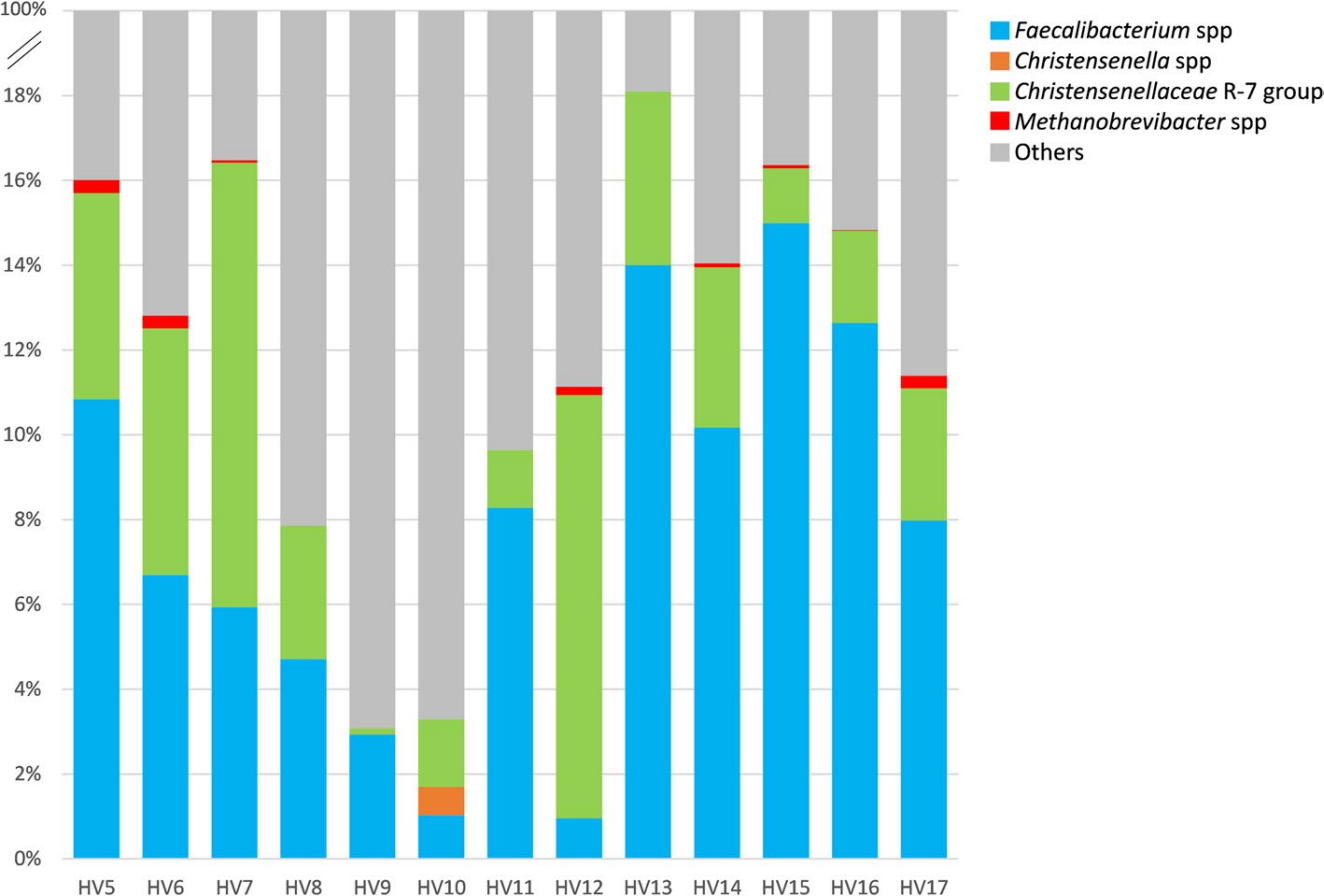
16S rRNA gene amplicons analysis of fecal samples used to sort and cultivate *F. prausnitzii* (HV5 to HV9) or *C. minuta* (HV10 to HV17). Only the genera with which the species we focused on in this study are affiliated are shown for clarity

Based on the results observed with pure cultures of *F. prausnitzii* collection strains, we chose to combine LIVE/DEAD™ staining with specific polyclonal antibodies to perform sorting and cultivation experiments from frozen fecal material. In this series of experiments with fecal samples collected from 5 healthy volunteers, Live (i.e., SYTO 9-positive and PI-negative) bacteria ranged from 27.5 to 36.5%. Similarly to what was observed in preliminary experiments, auto-fluorescent bacteria accounted for 0.22 to 1.18% of the events for 3 of the 5 volunteers when exciting with the 405 nm laser (Fig. 7 and Table 1). Interestingly, these 3 volunteers were also those for which OTUs affiliated to the *Methanobacteriaceae* family were detected in the 16S rRNA gene amplicon repertoire (Fig. 8). As for preliminary experiments, we did not observe any significant auto-fluorescence when exciting with the Red 640 nm laser (data not shown) and there was only little staining with the pre-immune antibodies conjugated with Alexa Fluor™ 647 (Table 1).

**Table 1:**
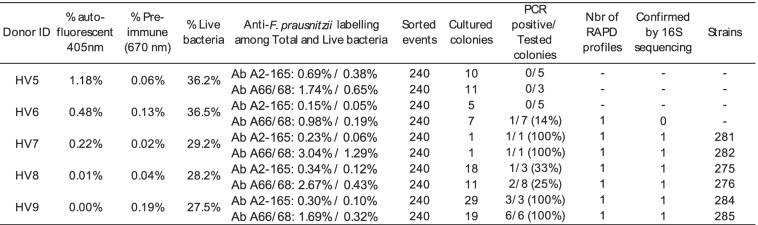
Results of FCM analysis, sorting and cultivation when targeting *F. prausnitzii* in samples HV5 to HV9

The percentage of bacteria individually stained with the anti-*F. prausnitzii* A2-165 antibodies represented up to 0.69% of total bacteria for HV5, falling to 0.38% when taking into account only live bacteria (Table 1). Percentages were higher for anti-*F. prausnitzii* ATCC 27766 +27768 antibodies, with up to 3.04% of total and 1.29% of live bacteria being stained for HV7.

For every fecal sample, 240 events stained with anti-*F. prausnitzii* A2-165 or anti-*F. prausnitzii* ATCC 27766 + ATCC 27768 antibodies were sorted and plated on mGAM-CRI plates, resulting in variable numbers of colonies depending on the fecal samples and on the gated regions. Forty-two colonies presenting morphologies compatible with *F. prausnitzii* (i.e., exclusion of large colonies that appeared in less than 48 h) were screened with species-specific primers, resulting in a total of 10 PCR-positive colonies isolated from 4 different donors in the Ab 66/68-gated events and 5 PCR-positive colonies isolated from 3 different donors in the Ab A2-165-gated events (Table 1). To assess the heterogeneity of the new isolates, we screened the colonies that appeared positive for species specific PCR using the RAPD, which allows discrimination of closely related strains of the same species [25]. We were able to distinguish 7 isolates presenting different RAPD profiles that were further analyzed using 16S rRNA gene sequencing to confirm their identity. Six of seven isolates were confirmed as *F. prausnitzii* whereas the unique isolate collected from HV6 was assigned as *Ruthenibacterium lactatiformans*, which belongs to the *Oscillospiraceae* family along with *F. prausnitzii* (Additional files 6 and 7).

Use of polyclonal antibodies for sorting and cultivation of new *C. minuta* strains from fecal material

**Fig. 9:**
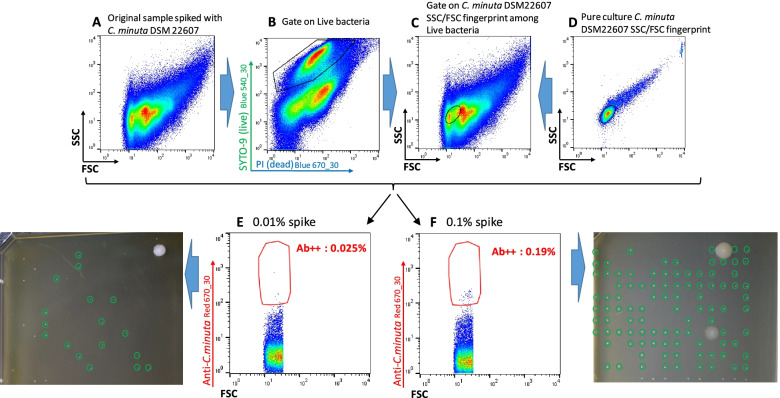
Development and validation of the *C. minuta* enrichment and sorting strategy. Original fecal sample was spiked with 0.1% or 0.01% *C. minuta* DSM 22607 (**A**). Bacteria considered as live after staining with the LIVE/DEAD™ *Bac*Light™ Bacterial Viability Kit were selected (**B**) and among them only events presenting FSC/SSC values similar to those of *C. minuta* DSM 22607 (as determined in panel **D**) were considered (**C**, black circle). Bacteria stained with anti-*C. minuta* antibodies were then selected from these regions and directly sorted on cultivation plates (**E** and **F**, antibodies-stained frequencies relate to total events). Colonies circled in green tested positive for *C. minuta*-specific PCR. These experiments were performed once for each spiked dilution

Since *C. minuta* is usually present in only very low amounts in fecal material compared to *F. prausnitzii* [6, 16], we first performed an experiment in which *C. minuta* was spiked in different amounts for better delineation of the sorting gate. Taking advantage of the very small size of cells from this bacterial species, we adjusted the gating strategy that consisted in selecting antibodies-stained bacteria among Live bacteria presenting FSC/SSC parameters similar to those of *C. minuta* DSM 22607 (Fig. 9). Twenty-five of 30 (83%) and 105 of 107 (98%) colonies recovered from the 0.01% and 0.1% spiked material, respectively, were confirmed as *C. minuta* by species-specific PCR, thus validating the selection strategy. We therefore used it to isolate *C. minuta* strains from fresh fecal samples from 8 healthy volunteers (HV10‑HV17). As expected, events that potentially correspond to *C. minuta* were scarce, ranging between nearly 0 and 1.0% of the Live bacteria among different samples. Colonies with *C. minuta*-compatible morphology (Fig. 9) that developed 48 to 72 h after plating were subjected to PCR analysis using species-specific primers. Between 13 and 64% of these colonies from 4 out of 8 samples were indeed positive in this assay (Table 2). Ten different RAPD profiles were observed but three of the isolates actually corresponded to species other than *C. minuta*, as demonstrated by partial 16S sequencing. We therefore ended with 7 different *C. minuta* isolates collected from 3 different donors and presenting 6 different RAPD profiles (Additional files 8 and 9). It should be noted that 16S gene amplicon repertoire sequencing detected *C. minuta* only for HV10 and HV17 fecal samples, representing 0.67 and 0.01% of OTUs, respectively (Fig. 8).

**Table 2:**
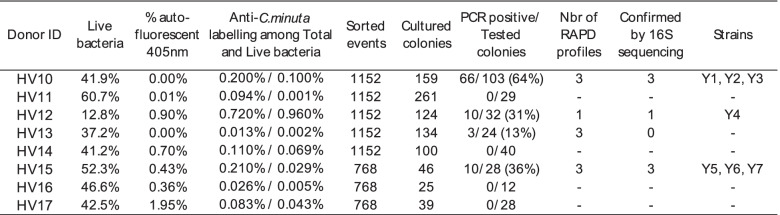
Results of FCM analysis, sorting and cultivation when targeting *C. minuta* in samples HV10 to HV17

#### Correlating sequencing results with 405 nm auto-fluorescence results

**Fig. 10:**
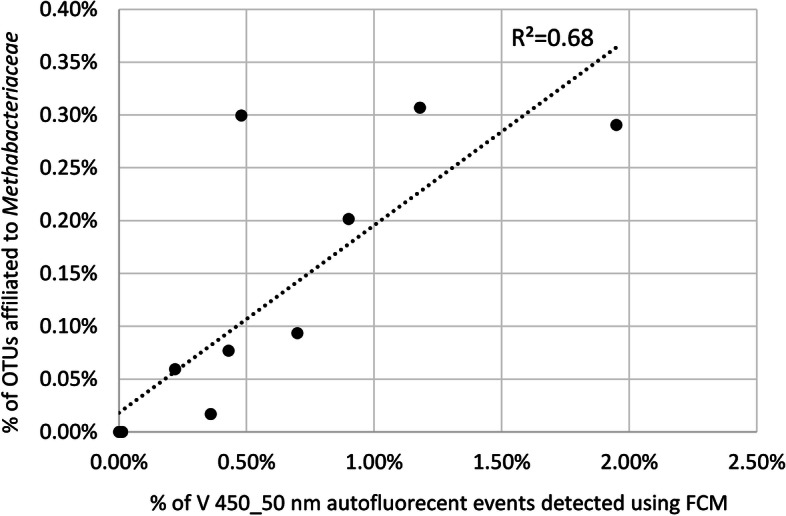
Correlation between relative abundance of autofluorescent events detected in the 450/50 nm channel when exciting with the 405 nm laser and relative abundance of *Methanobacteriaceae* calculated from 16S rRNA-encoding genes sequencing results for donors HV5 to HV 17. *P* value calculated with the Pearson method is 5.1 × 10^−4^

In order to better assess the relevance of auto-fluorescence measurement for detecting archaea, we compared relative abundance of auto-fluorescent events detected in cytometry to relative abundance of *Methanobacteriaceae* calculated from 16S rRNA-encoding genes sequencing results for the 13 donors (HV5 to HV 17) for which both data sets were available. This resulted in a *R*^2^ value of 0.68, with a systematic under-estimation of sequencing compared to FCM results (Fig. 10).

## Discussion

The recent description of FCM and bacterial cell sorting under anaerobic conditions [23] prompted us to explore this technology for targeted enrichment and culture of species of interest from fecal material. We first focused on the commensal species *F. prausnitzii* which is usually found in significant numbers in fecal samples, but is very sensitive to oxygen exposure. A first batch of antibodies was raised against the type strain A2-165 that belongs to *F. prausnitzii* phylogroup IIb, and a second batch was raised against a mix of the closely related strains ATCC 27766 and ATCC 27768 that belong to phylogroup I recently proposed as “Faecalibacterium moorei” [10]. Interestingly, polyclonal antibodies generated with type strains from one phylogroup presented only limited reactivity against type strains of the other phylogroup. This could be due to lower affinity binding, or to the limited presence of common epitopes. In addition, the presence of extracellular compounds that mask specific epitopes cannot be excluded [26]. The presence of such an extracellular matrix may depend on the growth phase, which could also explain the observed variations in staining efficiency against strain A2-165. In a similar way, polyclonal antibodies generated using type strain *C. minuta* DSM 22607 were only poorly reactive or even completely unreactive against type strains of “C. massiliensis” and “C. timonensis,” respectively. Both for the three *F. prausnitzii* strains and the *C. minuta* strain, bacteria sorted after antibody staining remained cultivable and LIVE/DEAD™ staining correlated well with cultivability, with only a few colonies obtained from bacteria stained by PI. We recently reported similar cultivability results after LIVE/DEAD™ staining for a limited number of anaerobic commensal species, suggesting that this labeling has real value for enriching anaerobic commensal bacteria to be sorted for cultivation [27]. However, one should remember that exceptions have also been reported [28] and that staining efficacy can potentially be affected if bacteria form endospores, which is the case for a variety of gut commensal species [29].

Since the ultimate goal of the sorting experiments was to cultivate stained bacteria rather than to measure their relative abundancy in the samples, we chose to use a stringent gating strategy that was common to all samples. Sorting and sequencing experiments confirmed the good species specificity of polyclonal antibodies directed against the two *F. prausnitzii* phylogroups, for which nonspecific enrichment was almost absent. The fact that we were not able to culture *F. prausnitzii* strains from fecal samples collected on HV5 and HV6 for which *F. prausnitzii* OTUs were detected by sequencing can be due to the specificity of the two antibodies, which probably do not cover the whole variety of possible strains. Since stained events were detected with both antibodies especially for HV5, another explanation could be that some strains require specific nutrients that were not present in our complemented mGAM medium. Concerning the two pairs of *F. prausnitzii* strains that were isolated from donors HV7 (strains 281 and 282) and HV8 (strains 275 and 276) using the two different polyclonal antibodies, it was interesting to note that they presented different but still related RAPD profiles (Additional Fig. 6) but that they presented 100% 16S rRNA-encoding gene identities with each other (Additional Fig. 7). Whether differences observed in RAPD profiles reflect evolution of one commensal strain through gain and/or loss or several genes [30] or are due to technical biases will be confirmed when sequencing the complete genomes. These results also call into question the specificity of our polyclonal antibodies. It cannot be excluded that phylogroup-specificity observed with polyclonal antibodies directed against type strains will be challenged by new strains that will react with both antibodies.

Autofluorescence of methanogenic archaea has already been reported: it is due to the redox cofactor F_420_ and has been proved useful for fast and reliable quantification of methanogenic archaea in biogas digesters using flow cytometry [31]. Although performed with a limited number of samples, our work tends to confirm that it could also be used to monitor methanogenic archaea in fecal samples. This could be of interest for the development of microbiota-based biomarkers since methanogenic archaea are considered major contributors to carbohydrate metabolism and their absence or presence in various amounts has been reported to be associated with several phenotypes, including severe acute malnutrition [32] or a lean phenotype [33, 34], to mention just a few. Interestingly, the presence of members of the *Christensenellaceae* family in the gut microbiota was reported to be associated with the presence of methanogenic archaea [17], with both groups being late colonizers of the gut ecosystem [35], which could be due to syntrophy via interspecies hydrogen transfer between *Christensenella* and *Methanobrevibacter* species [36]. In our limited number of samples, we did not observe any correlation between the presence of *Christensenellaceae* and the presence of methanogenic archaea when evaluated by FCM or 16S repertoire sequencing. *C. minuta* has been reported as a keystone species that comprises on average 0.01% of the fecal microbiota [16]. This low abundancy could explain why culture studies that used non-specific methods to cultivate a large diversity of gut commensal species did not succeed at cultivating *C. minuta* strains [11], thus highlighting the need for methods that can enrich cultivated fractions with specific species of interest. This includes antibodies, but also a number of additional stains such as for instance fluorescent analogs of glucose that have recently been shown to be taken up by members of the *Christensenellaceae*-R7 group [37].

In conclusion, this proof-of-concept study confirms that FCM is well adapted for complex bacterial microbiota studies. When used in conjunction with appropriate staining and associated controls, it gives a general overview of microbiota composition and variations in longitudinal studies [38], including bacterial load which is an important piece of information [39]. In addition, the use of more specific staining such as antibodies is a promising strategy to target, sort, and cultivate species of interest from these complex ecosystems. Recent studies demonstrated that these antibodies can be generated using a reverse genomics approach [40], which opens important avenues since approximately 70% of the gut microbial species still lack cultured representatives [41]. Due to the lack of detailed knowledge of their reactivity, it remains difficult to use polyclonal antibodies such as those described in this study for an analysis of the relative abundance of specific species of interest. However, in the future, better characterized monoclonal antibodies or antibody cocktails may offer an interesting alternative to molecular biology-based methods for longitudinal monitoring of commensal species of interest.

This should be accompanied by specific technological developments in the field of FCM to allow simple, commercially available solutions enabling routine sorting experiments in controlled atmosphere conditions, which will be of strong interest for commensal bacteria but also for cellular biology applications necessitating oxygen conditions that are close to in vivo conditions [42, 43].

## Methods

### Bacterial strains and growth conditions

The following reference strains were used in this study: *Faecalibacterium prausnitzii* DSM-17677 (A2-165), ATCC-27766, and ATCC-27768, *Christensenella minuta* DSM 22607. They were cultured using modified Gifu Anaerobic Medium (mGAM, HyServe 05426) complemented with 30% bovine rumen, cellobiose (1 mg/ml), and inulin (1 mg/ml) with (plates) or without (broth) 1.5% agar (mGAM-CRI). All media and reagents were reduced for at least 48 h in the BACTRON600 (Sheldon) anaerobic chamber before use, and cultures were incubated at 37 °C in the chamber.

### Polyclonal antibodies

Rabbit polyclonal antibodies (pAb) were produced in New Zealand rabbits using a standard 53 days protocol (Covalab). Rabbits were immunized with a 50/50 mix of phylogenetically related strains *F. prausnitzii* ATCC-27766 and ATCC-27768, or pure cultures of *F. prausnitzii* A2-165 and *C. minuta* DSM 22607. At day 0, animals received intradermal injection of 0.5 ml of a 1 × 10^9^ suspension of heat-inactivated bacteria + 0.5 ml of complete Freund's Adjuvant. At day 14 and day 39, they received sub-cutaneous injection of 0.5 ml of a 2x10^9^ suspension of heat-inactivated bacteria + 0.5 ml of incomplete Freund's Adjuvant. Serum samples collected at day 39 were tested for immune-reactivity. In case of good reactivity (which was the case for *F. prausnitzii A2-165*), animals were sacrificed at day 53 for serum collection. If reactivity of day 39 sera was too low (*F. prausnitzii* ATCC 27766 + ATCC 27768 and *C. minuta* DSM 22607), animals were boosted at days 60, 74 and 88 with sub-cutaneous injection of 0.5 ml of a 2x10^9^ suspension of heat-inactivated bacteria + 0.5 ml of incomplete Freund’s adjuvant before sacrifice at day 108. After rabbit bleeding, sera were harvested and IgGs were purified on protein A and labeled with Alexa Fluor™ 647 or Alexa Fluor™ 405 using the protein labeling kit (Thermo Fisher Scientific) as recommended by the manufacturer. IgGs from a non-immunized rabbit were also coupled with Alexa Fluor™ 647 as negative control.

### Viability staining procedure

Viability staining was performed using the LIVE/DEAD™ BacLight™ Bacterial Viability Kit (SYTO 9/Propidium Iodide, Thermo Fisher Scientific) as recommended by the manufacturer. After staining, bacteria were washed in PBS and then analyzed within 30 min using an Influx® (Becton-Dickinson) cell sorter equipped with a 200 mW-488 nm laser, a 120 mW-640 nm laser, and a 100 mW-405 nm laser.

### Anaerobic sorting of single strains

The BD Influx® cell sorter used for anaerobic sorting has been described by Thompson et al. [23]. Briefly, anaerobic sorting was achieved by eliminating oxygen from the sort stream and cell deposition areas of the cell sorter by using a customized glove box. Paraffin oil was used to cover samples in both conditions to prevent oxygen entry to the sample during the transfer from the anaerobic chamber, where the samples were prepared, to the flow cytometer. Durations of the sorting steps were normalized to 30 min. Oxygen concentration in the glove box was monitored using a ToxiRAE PRO detector (RAE, France). For sorting experiments, reduced mGAM-CRI plates were transferred from the anaerobic chamber to the cell sorter glove box using sealed bags. Nitrogen was then injected and anaerobic sorting experiments were started when the oxygen concentration was measured below 0.7%. Analysis and sorting followed by cultivation of stained and unstained bacteria was used to evaluate the impact of the process on *F. prausnitzii* and *C. minuta* cultivability. Bacteria used for sorting experiments were anaerobically cultivated for 48 h at 37 °C on mGAM-CRI plates. One colony was then sub-cultivated in mGAM-CRI broth for 24 h at 37 °C. One milliliter of bacterial cultures were then washed in reduced PBS and diluted 1:100 before staining with LIVE/DEAD™ BacLight™ Bacterial Viability Kit as described above. Polyclonal antibodies generated using the mix of *F. prausnitzii* ATCC 27766 and ATCC 27768 bacteria were conjugated with Alexa Fluor™ 647 whereas those generated with *F. prausnitzii* A2-165 were conjugated with Alexa Fluor™ 405. Polyclonal antibodies generated using *C. minuta* DSM 22607 bacteria were conjugated with Alexa Fluor™ 647. Staining with 1/100th antibodies (final concentration: 10 μg/ml) and viability kit was performed in anaerobic conditions for 30 min in the dark and then bacteria were washed in reduced PBS before analysis. After washing, stained bacteria were suspended in reduced PBS containing 0.5 mg/l resazurin, 2.1 mM soldium sulfure, and 2.8 mM L-cystein HCl, and the suspensions were covered with 750 μl of paraffin oil to prevent oxygen exposure. The tubes were taken out of the anaerobic chamber and bacteria were analyzed and sorted within 30 min. Sorting speed was adjusted at 1000 events per second and four series of 1, 3, 10, 30, 100, 300, or 1000 events were sorted on one single spot for each tested condition. Once sorting experiments were achieved, plates were re-introduced in sealed bags and transferred in the anaerobic chamber where they were incubated at 37 °C for 2 to 3 days before observation. Percentages of recovery were calculated by taking into account the smallest number of sorted bacteria resulting in the growth of colonies visible to the naked eye. The following formula was then applied: percentage of recovery = (n/N × 1/B) × 100

n: number of colonies counted per row (or for 2 rows when only one bacterium was deposited)

N: number of sorted spots (8 for the first 2 rows for which only one bacterium was deposited per spot, 4 for the other rows for which higher numbers of bacteria were deposited)

B: number of bacteria sorted on each spot.

### Unstained and pre-immune controls

We performed control experiments by analyzing a series of 4 fecal frozen samples collected from healthy volunteers (HV) 1 to 4. Samples were left unstained to evaluate potential auto-fluorescence, or stained with pre-immune antibodies conjugated with Alexa Fluor™ 647 dye to evaluate non-specific staining. The same 4 samples were also stained with both anti-*F. prausnitzii* antibodies in a separate tube. Briefly, washed bacteria were incubated in PBS with SYTO 9/PI added with 1/100th conjugated antibodies dilution (final concentration: 10 μg/ml) for 30 min. Bacteria were then washed once with reduced PBS, covered with paraffin oil to protect them from oxygen and analyzed by flow cytometry.

### Sorting and DNA extraction from antibodies-enriched fractions

To test the specificity of the *F. prausnitzii* antibodies, we sorted antibodies-stained fractions collected from the pool of frozen fecal samples HV2 and HV4. Bacteria stained by the polyclonal antibodies directed against *C. minuta* being almost undetected in the mix, we decided to spike the same pool of 2 fecal samples with *C. minuta* DSM 22607 at approx. 2% relative to the bacterial counts measured by flow cytometry. This allowed sorting of sufficient material for subsequent sequencing and evaluation of antibodies specificity. Events presenting auto-fluorescence when exciting with the 405 nm laser were also sorted for further identification. One million events were sorted for each of the 3 antibodies as well as for the auto-fluorescent events. DNA was extracted from the sorted cells using the mericon™ DNA Bacteria Kit (QIAGEN) with adjustments as follows. Bacterial cell pellets were resuspended in 20‑40 μl Fast Lysis Buffer, depending on the volume of the pellet, by brief, vigorous vortexing. The samples were placed into a thermal shaker (800 rpm) set to 100 °C for 10 min. Samples were then allowed to cool at room temperature for 2 min, before centrifugation (13,000 × *g*, 5 min). 20‑40 μl of the supernatant were transferred to a 1.5 ml microcentrifuge tube and purified using the NucleoSpin™ Gel and PCR Clean-up Kit (Macherey-Nagel) according to manufacturer’s instructions.

### Library preparation and 16S rRNA gene amplicon data analysis

For native fecal samples, library preparation and sequencing were performed with 24 ng template DNA as described in detail previously [44] using a robotized platform (Biomek400, Beckman Coulter). For the samples extracted with the mericon™ DNA Bacteria Kit (QIAGEN), 1‑8 μL template DNA were used for PCR. The V3-V4 region of 16S rRNA genes was amplified in duplicates for 25 cycles with DNA from fecal samples, or with 35 cycles with DNA from cell sorted samples, following a two-step protocol [45] using primers 341F-785R [46]. After purification using the AMPure XP system (Beckman Coulter), sequencing was carried out with pooled samples spiked with 25% (v/v) PhiX standard library in paired-end modus (PE300) using a MiSeq system (Illumina, Inc.) according to the manufacturer’s instructions. Raw reads were processed using an in-house developed pipeline (www.imngs.org) [47] based on UPARSE [48]. In brief, sequences were demultiplexed and trimmed to the first base with a quality score < 3. Pairing, chimera filtering and operational taxonomic units (OTUs) clustering (97% sequence identity) was done using USEARCH 11.0 [49]. Sequences with less than 350 and more than 500 nucleotides and with an expected error > 2 were excluded from the analysis. Remaining reads were trimmed by ten nucleotides on each end to avoid GC bias and non-random base composition. Only OTUs occurring at a relative abundance > 0.25% in at least one sample were kept. To highlight differences in specificities of the antibodies raised against *F. prausnitzii* that includes different phylogroups, a zero-radius approach [24] using the UNOISE algorithm (USEARCH 11.0) [50] was chosen, increasing the taxonomic resolution with which the molecular species in sorted bacterial populations were delineated. Sequence alignment and taxonomic classification was conducted with SINA v1.6.1, using the taxonomy within the SILVA release 128 [51]. Downstream analysis was performed in the R programming environment using Rhea (https://lagkouvardos.github.io/Rhea/) [52]. Normalization of OTU and ZOTU tables was performed by dividing through sample size and subsequent multiplication by the number of reads in the smallest sample, to account for differences in sequencing depth.

### Isolation of bacterial DNA from stool samples

DNA was isolated using a modified protocol according to Godon et al. [53]. Snap frozen samples were mixed with 600 μl stool DNA stabilizer (Stratec biomedical), thawed, and transferred into autoclaved 2-ml screw-cap tubes containing 500 mg 0.1 mm-diameter silica/zirconia beads. Next, 250 μl 4 M guanidine thiocyanate in 0.1 M Tris (pH 7.5) and 500 μl 5% N-lauroyl sarcosine in 0.1 M PBS (pH 8.0) were added. Samples were incubated at 70 °C and 700 rpm for 60 min. A FastPrep® instrument (MP Biomedicals) fitted with a 24 × 2 ml cooling adaptor filled with dry ice was used for cell disruption. The program was run 3 times for 40 s at 6.5 M/s. After each run, the cooling adapter was refilled with dry ice. An amount of 15 mg polyvinylpyrrolidone (PVPP) was added and samples were vortexed, followed by 3 min centrifugation at 15.000 × g and 4 °C. Approximately, 650 μl of the supernatant were transferred into a new 2 ml tube, which was centrifuged again for 3 min at 15.000 × g and 4 °C. Subsequently, 500 μl of the supernatant was transferred into a new 2 ml tube and 50 μg of RNase was added. After 20 min at 37 °C and 700 rpm, gDNA was isolated using the NucleoSpin® gDNA Clean-up Kit from Macherey-Nagel. Isolation was performed according to the manufacturer’s protocol. DNA was eluted from columns twice using 40 μl elution buffer and concentration was measured with NanoDrop® (Thermo Scientific). Samples were stored at −20 °C before being processed as described above for 16S repertoire analysis.

### Anaerobic sorting and cultivation from fecal material

Fecal samples were collected at home by healthy volunteers and immediately transferred in plastic pouches that were then sealed after adding an AnaeroGen™ sachet (Oxoid). Samples were shipped to the lab within 2 h at ambient temperature. All subsequent steps were performed in the anaerobic chamber. For each sample, 1 g of fecal material was suspended in 10 ml PBS and homogenized by vortexing using 2.4 mm glass beads. Suspensions were then 10-fold diluted in PBS and filtered through a 70-μm cell strainer (Biologix). After an additional 100-fold dilution in PBS, bacteria were numbered by FCM using counting beads (LIVE/DEAD™ BacLight™ Bacterial Viability and Counting Kit, Thermo Fisher Scientific). LIVE/DEAD™ staining was used to select live bacteria whereas polyclonal antibodies were used to enrich with target bacterial species. Staining was performed in anaerobic conditions for 30 min in the dark and then bacteria were washed in reduced PBS before analysis. After washing, stained bacteria were suspended in reduced PBS and the suspensions were covered with 500 μl of paraffin oil to prevent oxygen exposure. The tubes were taken out of the anaerobic chamber and bacteria were analyzed and sorted within 30 min. Bacteria were gated based on FSC/SSC parameters. Among them, live ones were selected according to SYTO 9/PI fluorescence and 240 (*F. prausnitzii*) to 1152 (*C. minuta*) events collected from the antibodies-stained gates were sorted on mGAM-CRI plates. Plates were then incubated for 5 days at 37 °C in anaerobic conditions. Colonies were then visually selected based on morphologies that were compatible with the target species: small, flat colonies encrusted in the agar plates for *F. prausnitzii*, and tiny bulging colonies for *C. minuta*. They were then screened using previously described PCR primers: Fprau 02 (5′-GAG CCT CAG CGT CAG TTG GT-3′) and Fprau 07 (5′-CCA TGA ATT GCC TTC AAA ACT GTT-3′) for *F. prausnitzii* [7], Yso F (5′-CCC ACC AAG TCAA CGA TGG G-3′), and ChrisM-R1 (5′-CCC TCT CCT GTA CTC AAG TC-3′) for *C. minuta* [54] and this study.

### Identification of cultured isolates

To distinguish potentially different strains isolated from the same sample, Random Amplified Polymorphic DNA (RAPD) analysis was conducted. Briefly, candidate *F. prausnitzii* or *C. minuta* colonies were subcultivated on mGAM plates and then several colonies were resuspended in 100 μl DNA-free water and DNA was isolated using InstaGene™ Matrix (BioRad) kit according to the manufacturer guidelines. This DNA preparation served as a template for PCR reaction using primer D9355 for *F. prausnitzii* candidates and primer D14307 for *C. minuta* candidates, and a PCR program described by Akopyanz et al. [25]. One isolate corresponding to each specific RAPD profile was then chosen for further identification by sequencing the almost complete 16S rRNA-encoding genes.

For phylogenetic analysis, 16S rRNA-encoding genes were aligned using Muscle [55] integrated in MEGA7 [56] with default parameters. The phylogenetic tree was inferred using the Maximum Likelyhood method based on the Kimura 2-parameters model with 1000 bootstrap replicates [19].

## Supplementary Information

**Additional file 1. **Unstained, pre-immune-stained and antibodies-stained controls for *F. prausnitzii* strains A2-165 (phylogroup IIb), ATCC 27766 (phylogroup I) and ATCC 27768 (phylogroup I).

**Additional file 2. **Unstained, pre-immune-stained and antibodies-stained controls for *C. minuta DSM 22607,* ‘C. massiliensis’ *DSM 102344 and* ‘C. timonensis’ *DSM 102800.*

**Additional file 3. **Gates used to select, sort and culitvate *F. prausnitzii* and *C. minuta* bacteria after staining with the LIVE/DEAD™ kit and with the specific antibodies.

**Additional file 4. **Preliminary FCM controls. (A) events gated as bacteria, (B) auto-fluorescence of fecal material in the Violet 450/50 nm and Red 670/30 nm channels, (C) potential staining with polyclonal antibodies collected from pre-immune serum and conjugated with Alexa Fluor™ 647, and (D) staining with anti-*F. prausnitzii* A2-165 and anti-*F. prausnitzii* ATCC 27766 + 27768 antibodies conjugated with Alexa Fluor™ 405 and Alexa Fluor™ 647, respectively.

**Additional file 5. **Gates that were used to sort and then sequence the 16S rRNA gene amplicon repertoire of bacteria stained with the *C. minuta*-antibodies.

**Additional file 6. **Random Amplified Polymorphism DNA profiles obtained with newly isolated *F. prausnitzii* strains using primer D9355 [25].

**Additional file 7. **Phylogenetic tree representing newly isolated *F. prausnitzii* strains. The phylogenetic tree was inferred from Muscle alignment of partial 16S rRNA-encoding gene sequences using the Maximum Likelihood method based on the Kimura 2-parameters model with 1,000 bootstrap replicates. Branch values < 50% are not displayed. The tree was built using reference sequences and outgroups described by [10]. Colonies aspects (bar: 0.5 cm) as well as Gram-stains (100x objective lens) are reported for the strains used in this study. Previously described strains with demonstrated anti-inflammatory activities are indicated with a red arrow. Strains highlighted in red were isolated with the polyclonal antibodies directed against *F. prausnitzii* ATCC 27766 + 27768, strains highlighted in violet were isolated with the polyclonal antibodies directed against *F. prausntizii* A2-165.

**Additional file 8. **Random Amplified Polymorphism DNA profiles obtained with newly isolated *C. minuta* strains using primer D14307 [25].

**Additional file 9. **Phylogenetic tree representing newly isolated *C. minuta* strains. The phylogenetic tree was inferred from Muscle alignment of partial 16S rRNA-encoding gene sequences using the Maximum Likelihood method based on the Kimura 2-parameters model with 1,000 bootstrap replicates. Branch values < 50% are not displayed. The tree was built using reference sequences and outgroups described in [57].

## Data Availability

16S gene amplicon sequencing data generated from fecal samples collected from healthy volunteers HV5-HV17 and from sorted fractions were deposited in Sequence Read Archive under accession number PRJNA748004. Partial 16S-rRNA encoding gene sequences of the new *F. prausnitzii* and *C. minuta* isolates described in this study are available under GenBank accession numbers MZ577583 to MZ577588, and MZ577589 to MZ577595, respectively.
