# Supplementary figures and images for: Species-targeted sorting and cultivation of commensal bacteria from the gut microbiome using flow cytometry under anaerobic conditions
Source: Microbiome. 2022 Feb 3;10:24. doi: 10.1186/s40168-021-01206-7 (PMC8812257; doi:10.1186/s40168-021-01206-7)

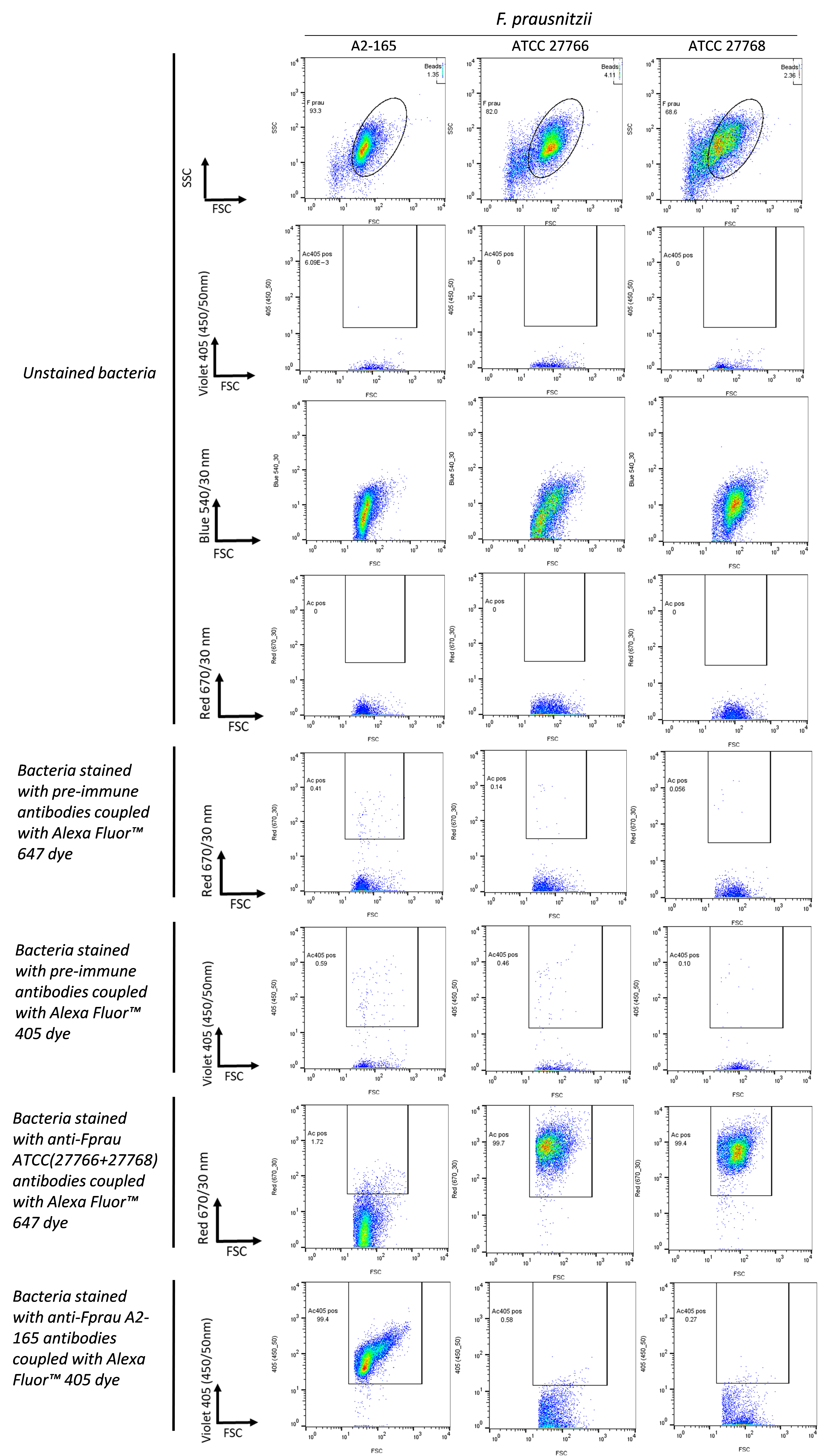

Supplement: Supplementary file 2 — Additional file 1. Unstained, pre-immune-stained and antibodies-stained controls for F. prausnitzii strains A2-165 (phylogroup IIb), ATCC 27766 (phylogroup I) and ATCC 27768 (phylogroup I). [file 40168_2021_1206_MOESM2_ESM.tif]

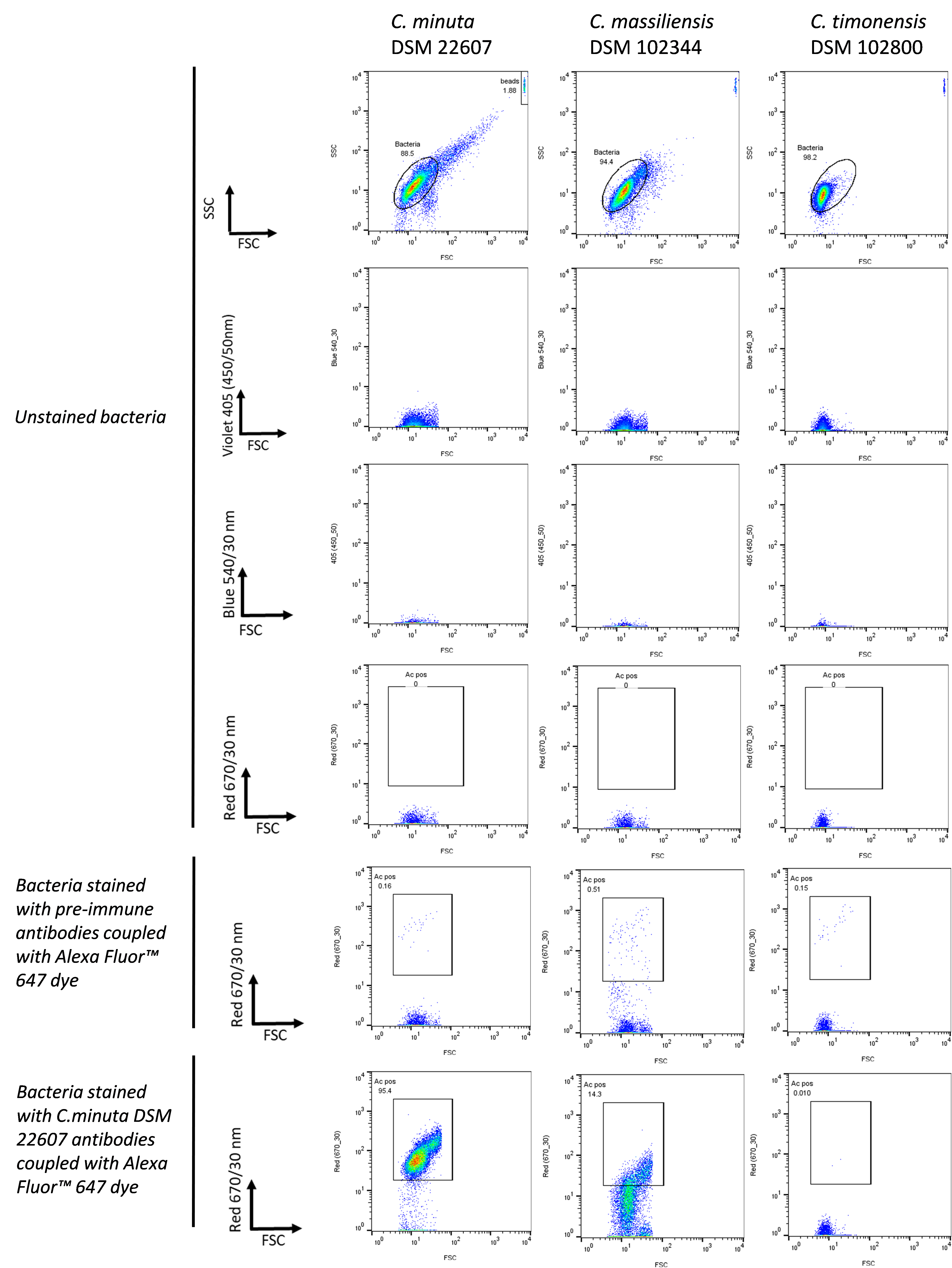

Supplement: Supplementary file 3 — Additional file 2. Unstained, pre-immune-stained and antibodies-stained controls for C. minuta DSM 22607, ‘C. massiliensis’ DSM 102344 and ‘C. timonensis’ DSM 102800. [file 40168_2021_1206_MOESM3_ESM.tif]

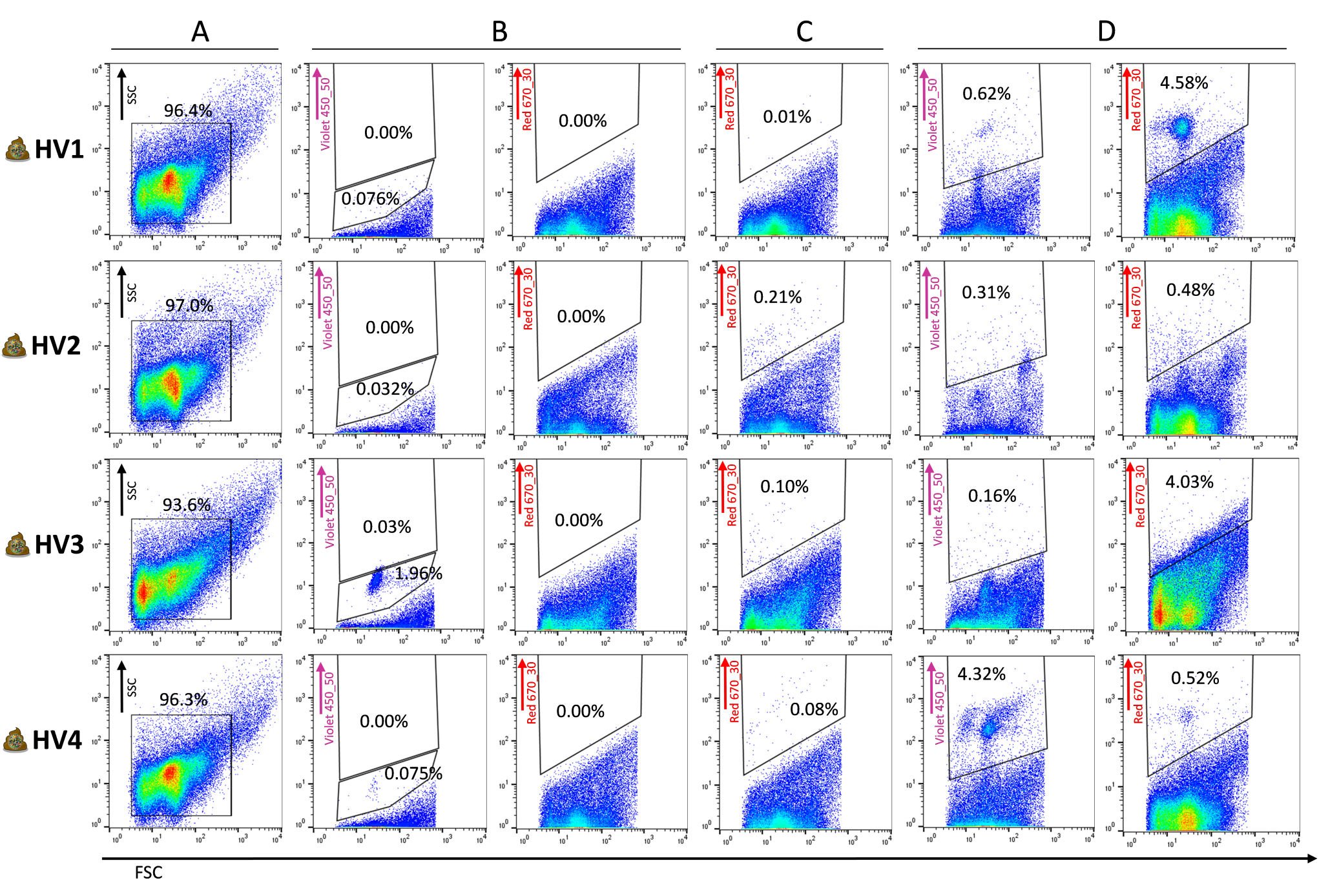

Supplement: Supplementary file 5 — Additional file 4. Preliminary FCM controls. (A) events gated as bacteria, (B) auto-fluorescence of fecal material in the Violet 450/50 nm and Red 670/30 nm channels, (C) potential staining with polyclonal antibodies collected from pre-immune serum and conjugated with Alexa Fluor™ 647, and (D) staining with anti-F. prausnitzii A2-165 and anti-F. prausnitzii ATCC 27766 + 27768 antibodies conjugated with Alexa Fluor™ 405 and Alexa Fluor™ 647, respectively. [file 40168_2021_1206_MOESM5_ESM.tif]

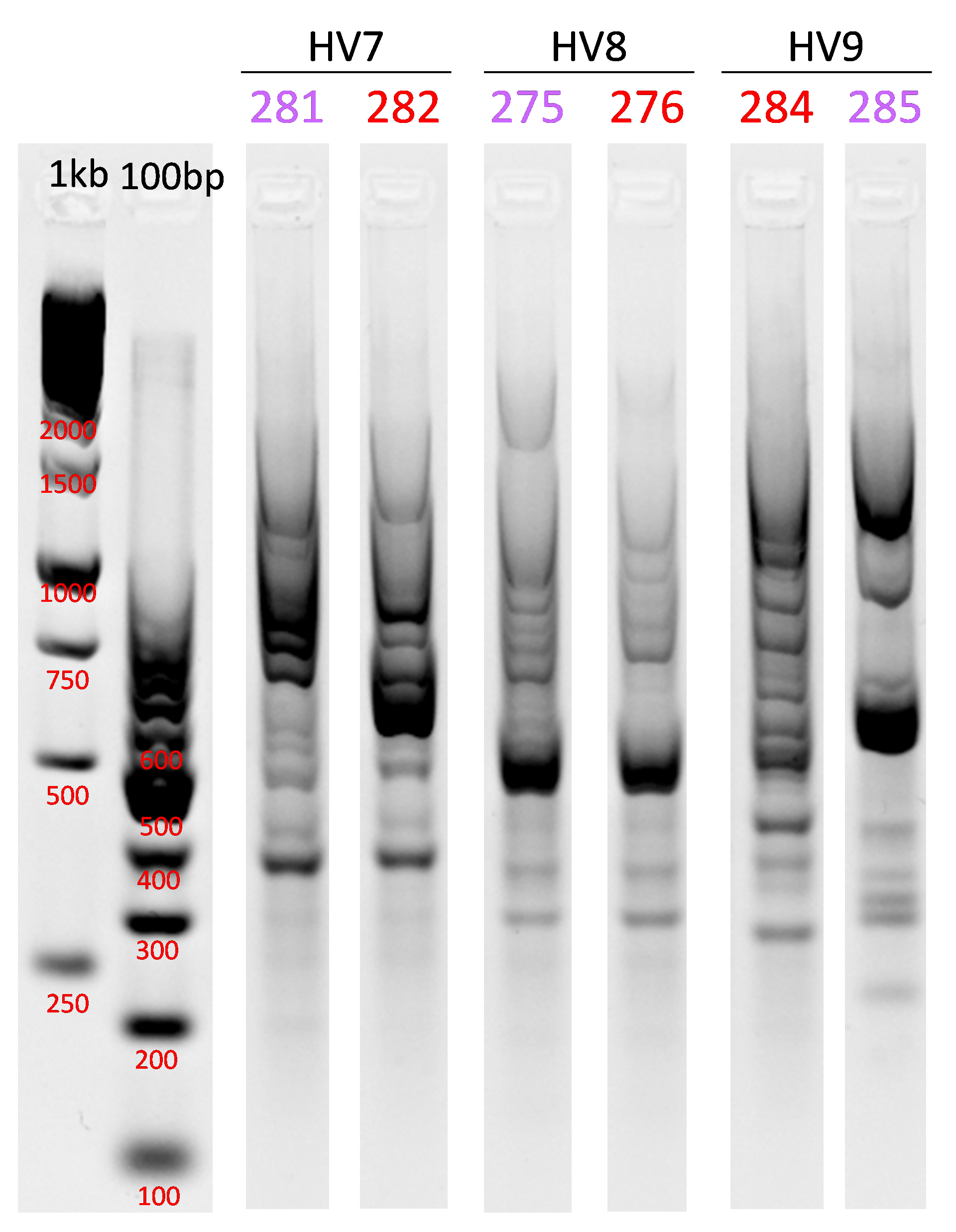

Supplement: Supplementary file 7 — Additional file 6. Random Amplified Polymorphism DNA profiles obtained with newly isolated F. prausnitzii strains using primer D9355 [25]. [file 40168_2021_1206_MOESM7_ESM.tif]

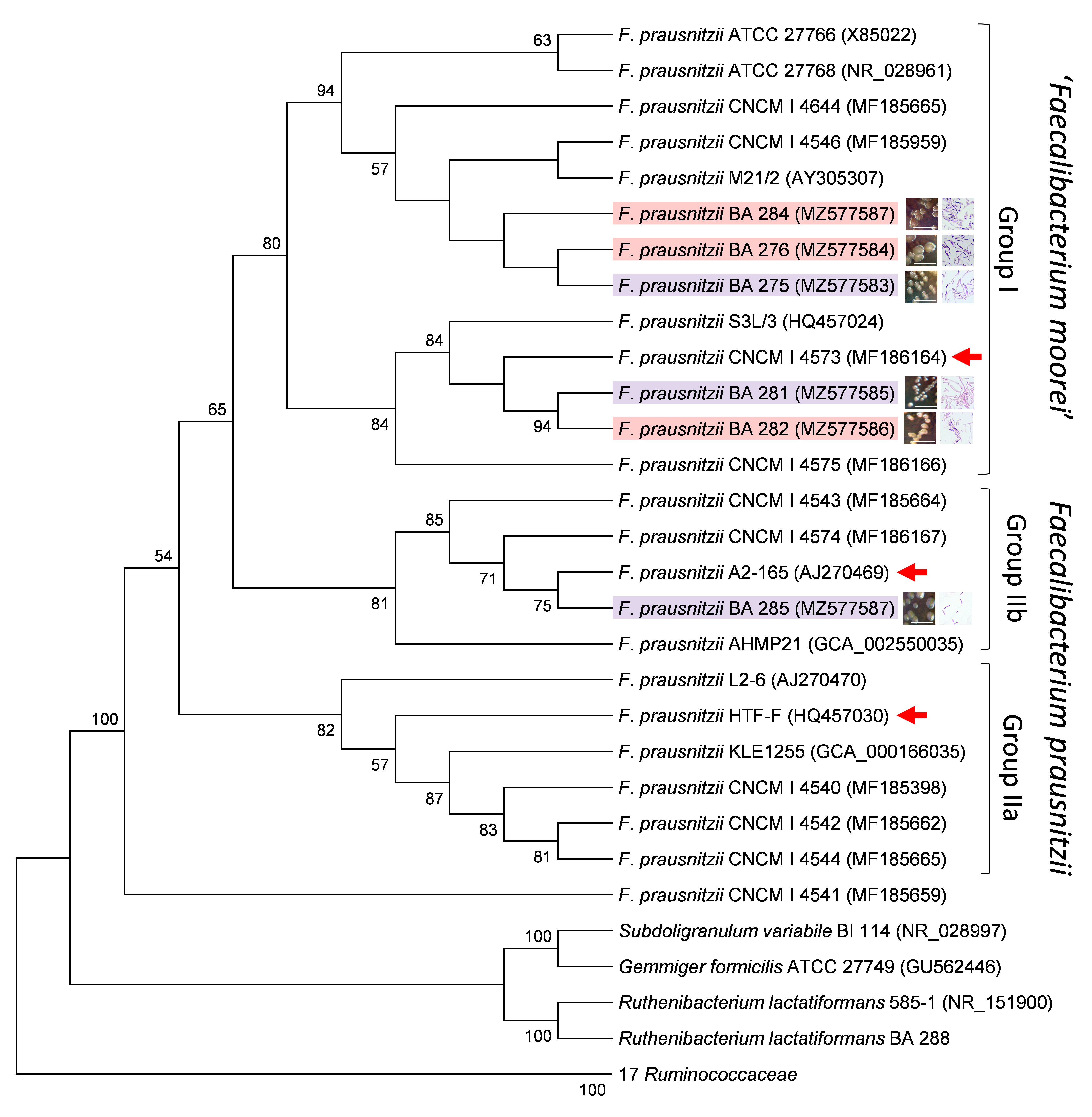

Supplement: Supplementary file 8 — Additional file 7. Phylogenetic tree representing newly isolated F. prausnitzii strains. The phylogenetic tree was inferred from Muscle alignment of partial 16S rRNA-encoding gene sequences using the Maximum Likelihood method based on the Kimura 2-parameters model with 1,000 bootstrap replicates. Branch values < 50% are not displayed. The tree was built using reference sequences and outgroups described by [10]. Colonies aspects (bar: 0.5 cm) as well as Gram-stains (100x objective lens) are reported for the strains used in this study. Previously described strains with demonstrated anti-inflammatory activities are indicated with a red arrow. Strains highlighted in red were isolated with the polyclonal antibodies directed against F. prausnitzii ATCC 27766 + 27768, strains highlighted in violet were isolated with the polyclonal antibodies directed against F. prausntizii A2-165. [file 40168_2021_1206_MOESM8_ESM.tif]

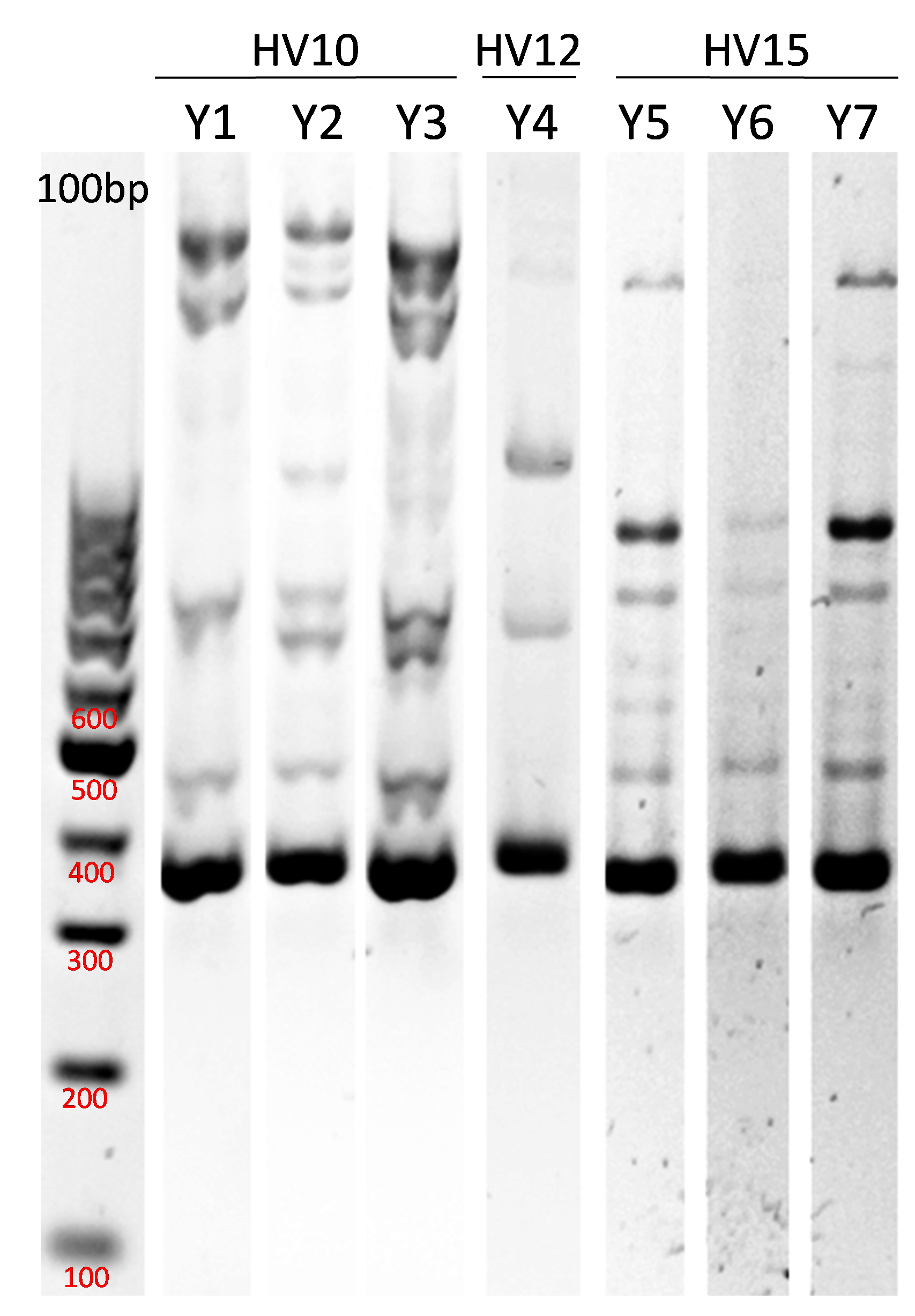

Supplement: Supplementary file 9 — Additional file 8. Random Amplified Polymorphism DNA profiles obtained with newly isolated C. minuta strains using primer D14307 [25]. [file 40168_2021_1206_MOESM9_ESM.tif]

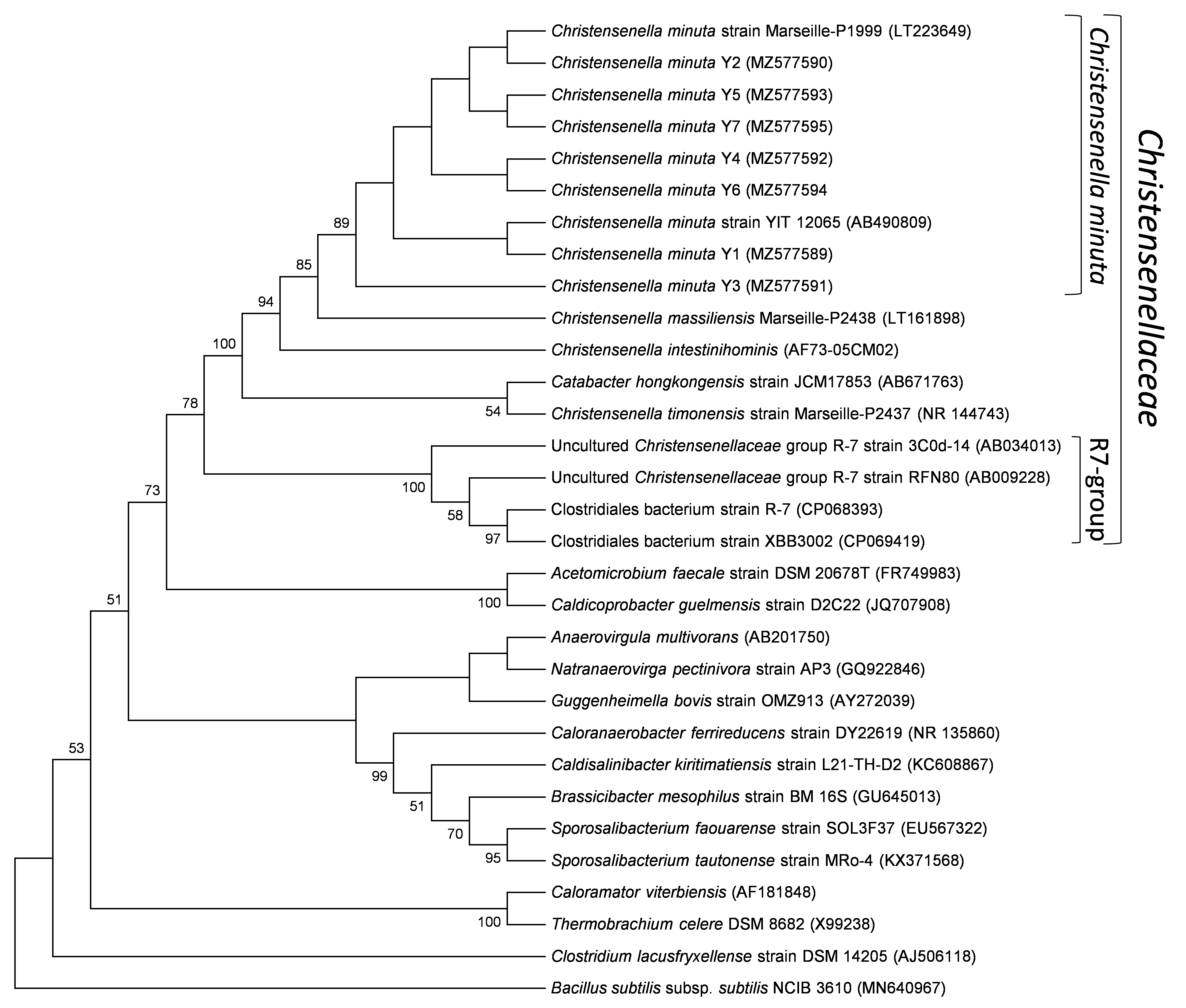

Supplement: Supplementary file 10 — Additional file 9. Phylogenetic tree representing newly isolated C. minuta strains. The phylogenetic tree was inferred from Muscle alignment of partial 16S rRNA-encoding gene sequences using the Maximum Likelihood method based on the Kimura 2-parameters model with 1,000 bootstrap replicates. Branch values < 50% are not displayed. The tree was built using reference sequences and outgroups described in [57]. [file 40168_2021_1206_MOESM10_ESM.tif]
