# Supplementary material for: Species-targeted sorting and cultivation of commensal bacteria from the gut microbiome using flow cytometry under anaerobic conditions
Source: Microbiome. 2022 Feb 3;10:24. doi: 10.1186/s40168-021-01206-7 (PMC8812257; doi:10.1186/s40168-021-01206-7)
Supplement: Supplementary file 4 — Additional file 3. Gates used to select, sort and culitvate F. prausnitzii and C. minuta bacteria after staining with the LIVE/DEAD™ kit and with the specific antibodies. [file 40168_2021_1206_MOESM4_ESM.pptx]

## Slide 1
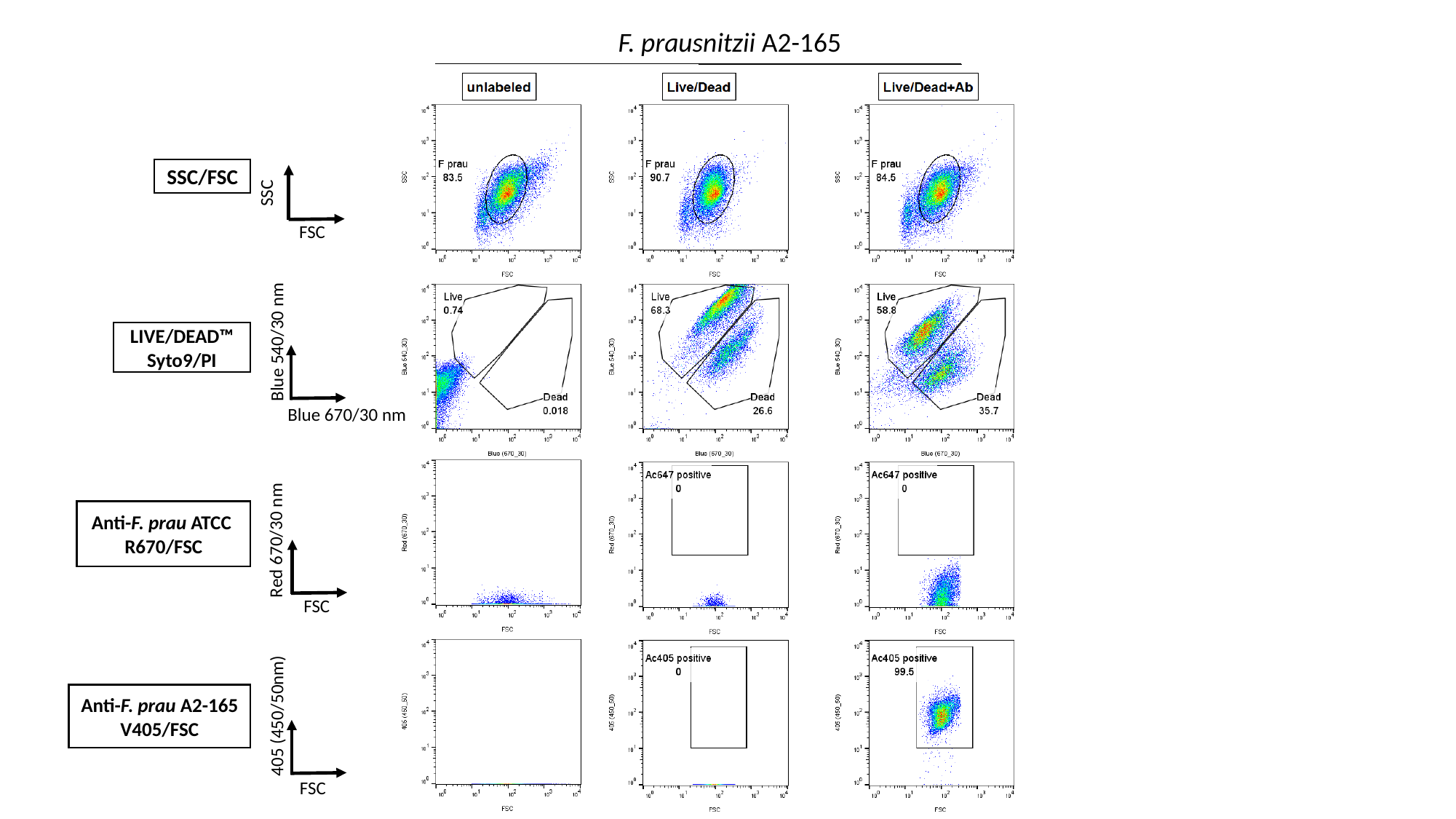

F. prausnitzii A2-165
SSC/FSC
SSC
FSC
LIVE/DEAD™
Syto9/PI
Blue 540/30 nm
Blue 670/30 nm
Anti-F. prau ATCC
R670/FSC
Red 670/30 nm
FSC
Anti-F. prau A2-165
V405/FSC
405 (450/50nm)
FSC

## Slide 2
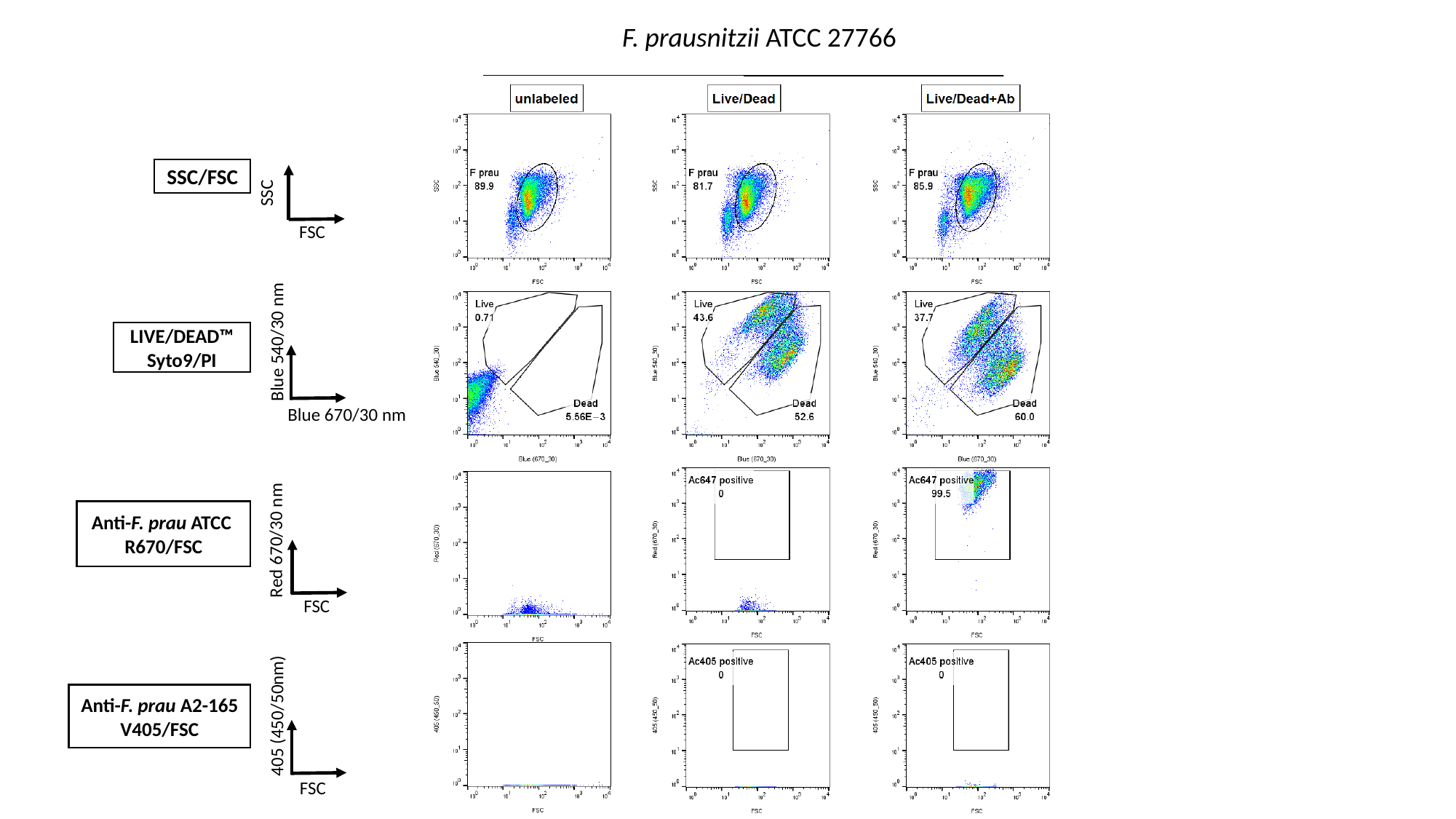

F. prausnitzii ATCC 27766
SSC/FSC
SSC
FSC
LIVE/DEAD™
Syto9/PI
Blue 540/30 nm
Blue 670/30 nm
Anti-F. prau ATCC
R670/FSC
Red 670/30 nm
FSC
Anti-F. prau A2-165
V405/FSC
405 (450/50nm)
FSC

## Slide 3
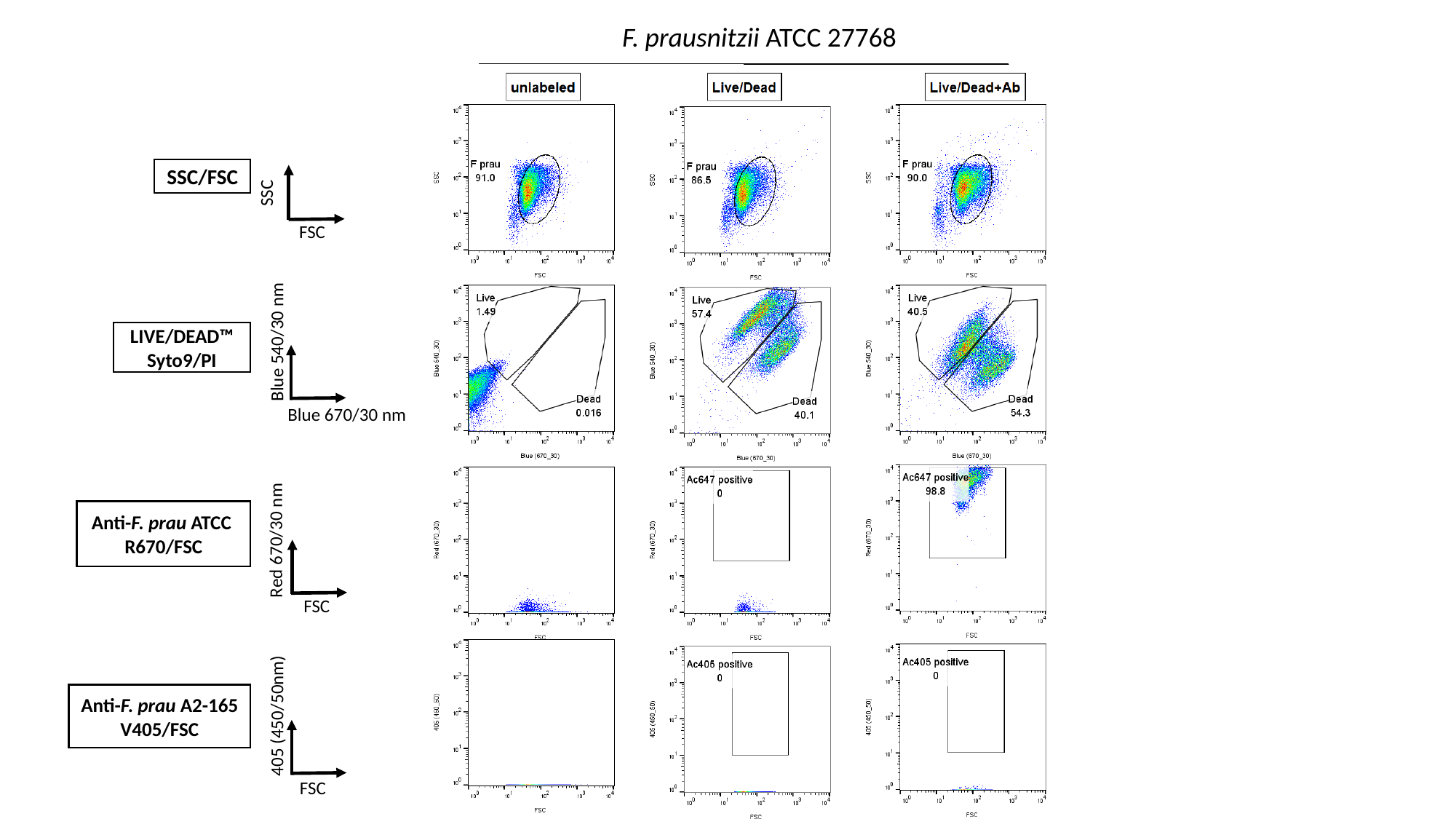

F. prausnitzii ATCC 27768
SSC/FSC
SSC
FSC
LIVE/DEAD™
Syto9/PI
Blue 540/30 nm
Blue 670/30 nm
Anti-F. prau ATCC
R670/FSC
Red 670/30 nm
FSC
Anti-F. prau A2-165
V405/FSC
405 (450/50nm)
FSC

## Slide 4
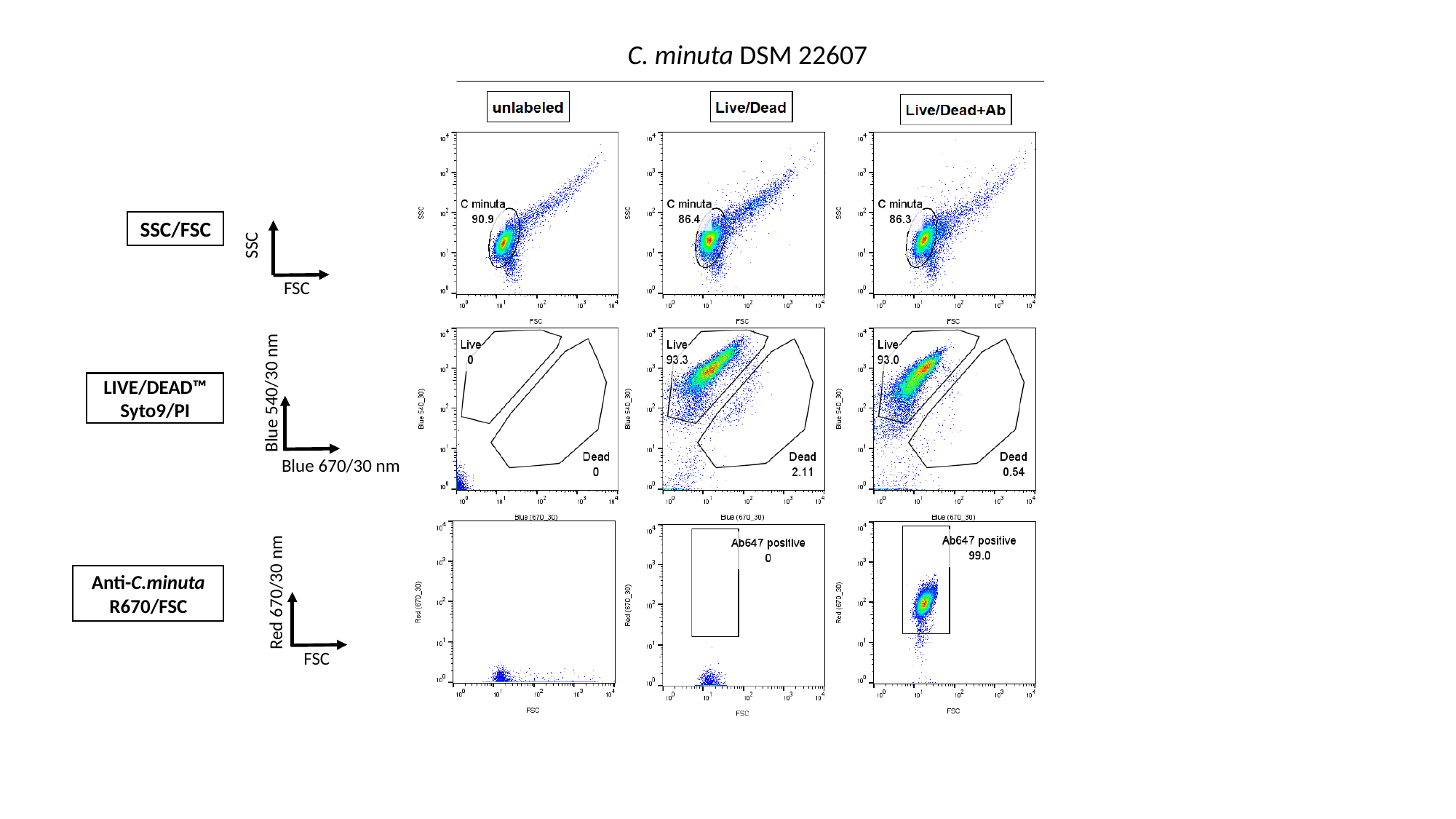

C. minuta DSM 22607
SSC/FSC
SSC
FSC
LIVE/DEAD™
Syto9/PI
Blue 540/30 nm
Blue 670/30 nm
Anti-C.minuta
R670/FSC
Red 670/30 nm
FSC
