# Supplementary material for: Species-targeted sorting and cultivation of commensal bacteria from the gut microbiome using flow cytometry under anaerobic conditions
Source: Microbiome. 2022 Feb 3;10:24. doi: 10.1186/s40168-021-01206-7 (PMC8812257; doi:10.1186/s40168-021-01206-7)
Supplement: Supplementary file 6 — Additional file 5. Gates that were used to sort and then sequence the 16S rRNA gene amplicon repertoire of bacteria stained with the C. minuta-antibodies. [file 40168_2021_1206_MOESM6_ESM.pptx]

## Slide 1
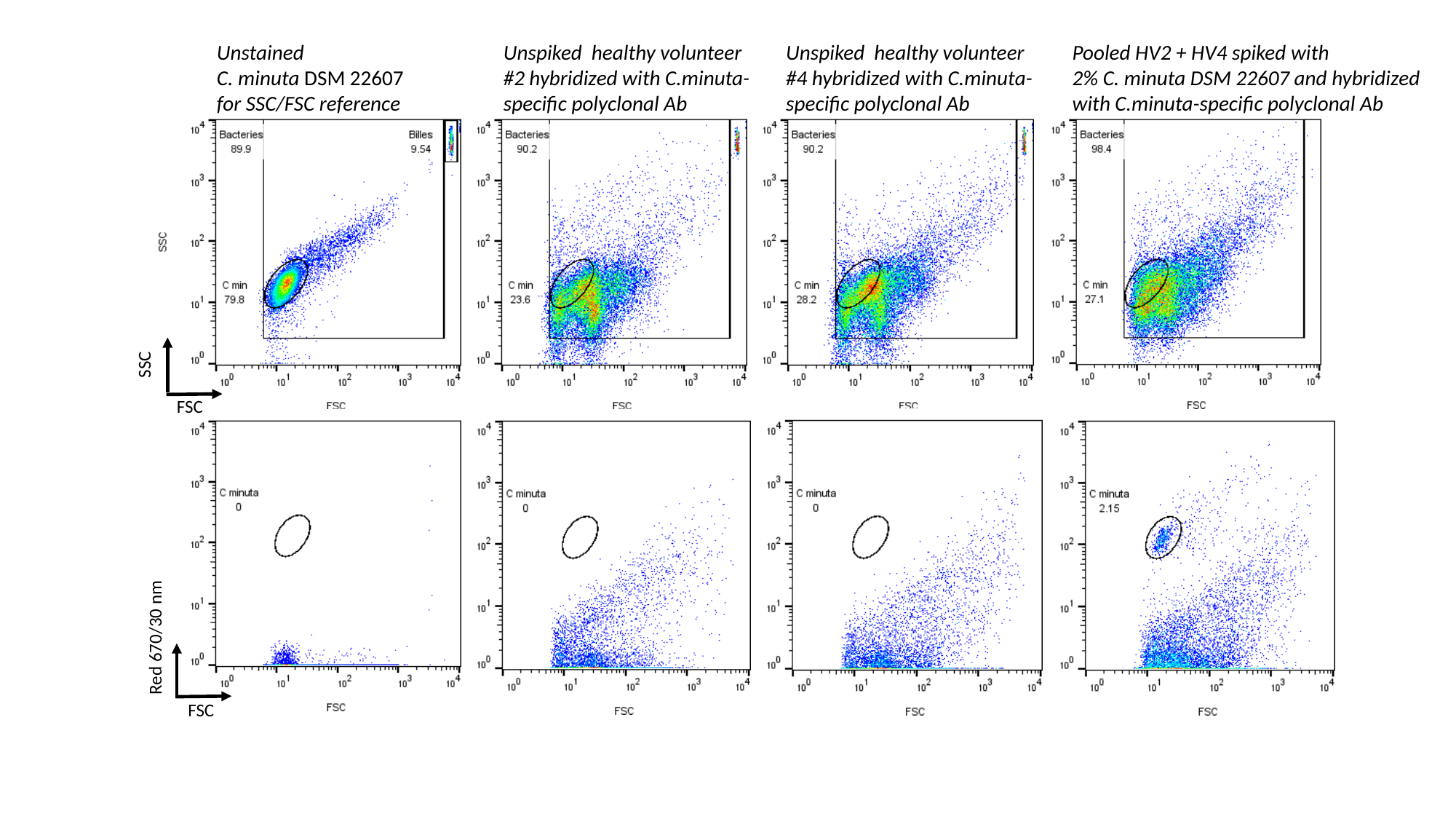

Unstained
C. minuta DSM 22607
for SSC/FSC reference
Unspiked healthy volunteer #2 hybridized with C.minuta-specific polyclonal Ab
Unspiked healthy volunteer #4 hybridized with C.minuta-specific polyclonal Ab
Pooled HV2 + HV4 spiked with
2% C. minuta DSM 22607 and hybridized with C.minuta-specific polyclonal Ab
SSC
FSC
Red 670/30 nm
FSC
